# Extracting Fluorescent Reporter Time Courses of Cell Lineages from High-Throughput Microscopy at Low Temporal Resolution

**DOI:** 10.1371/journal.pone.0027886

**Published:** 2011-12-15

**Authors:** Mike J. Downey, Danuta M. Jeziorska, Sascha Ott, T. Katherine Tamai, Georgy Koentges, Keith W. Vance, Till Bretschneider

**Affiliations:** 1 Molecular Organisation and Assembly in Cells, University of Warwick, Coventry, United Kingdom; 2 School of Life Sciences, University of Warwick, Coventry, United Kingdom; 3 Warwick Systems Biology Centre, University of Warwick, Coventry, United Kingdom; 4 Department of Cell and Developmental Biology, University College London, London, United Kingdom; Virginia Tech, United States of America

## Abstract

The extraction of fluorescence time course data is a major bottleneck in high-throughput live-cell microscopy. Here we present an extendible framework based on the open-source image analysis software ImageJ, which aims in particular at analyzing the expression of fluorescent reporters through cell divisions. The ability to track individual cell lineages is essential for the analysis of gene regulatory factors involved in the control of cell fate and identity decisions. In our approach, cell nuclei are identified using Hoechst, and a characteristic drop in Hoechst fluorescence helps to detect dividing cells. We first compare the efficiency and accuracy of different segmentation methods and then present a statistical scoring algorithm for cell tracking, which draws on the combination of various features, such as nuclear intensity, area or shape, and importantly, dynamic changes thereof. Principal component analysis is used to determine the most significant features, and a global parameter search is performed to determine the weighting of individual features. Our algorithm has been optimized to cope with large cell movements, and we were able to semi-automatically extract cell trajectories across three cell generations. Based on the MTrackJ plugin for ImageJ, we have developed tools to efficiently validate tracks and manually correct them by connecting broken trajectories and reassigning falsely connected cell positions. A gold standard consisting of two time-series with 15,000 validated positions will be released as a valuable resource for benchmarking. We demonstrate how our method can be applied to analyze fluorescence distributions generated from mouse stem cells transfected with reporter constructs containing transcriptional control elements of the Msx1 gene, a regulator of pluripotency, in mother and daughter cells. Furthermore, we show by tracking zebrafish PAC2 cells expressing FUCCI cell cycle markers, our framework can be easily adapted to different cell types and fluorescent markers.

## Introduction

Live cell fluorescent reporter-based techniques reveal the dynamics of gene expression under the control of different regulatory promoters, in individual cells and over periods of several days. Destabilized reporters with short half-lives of ∼30 minutes not only show when genes are turned on, but also how long expression lasts and possible periodic or random repetitions, either self-stimulated or induced. Single cell studies uncover the characteristics and effects of noise in transcriptional control by making it possible to synchronize temporal expression profiles *in silico* [1]–[3], contrary to population assays where individual responses are averaged out [4], [5]. Much progress has been made in high-throughput microscopy of tissue culture systems to study cells through several rounds of division [6], [7], with great potential to investigate differential gene expression in self-renewing and differentiating stem cells.

Commercial platforms are available that offer integrated setups containing a fluorescence microscope connected to a high resolution CCD camera with autofocus, a humidified incubator, liquid handling robots and computer systems allowing the automated imaging of thousands of cells [8]–[11]. A major limitation of current single cell approaches is, however, the identification and tracking of cells in time-series, both through cell divisions and in confluent cultures.

### Identifying cells using nuclear markers

The requirement to generate multiple clonal cell lines containing targeted insertion of reporter plasmids limits the use of stable transfections in large scale synthetic biology promoter studies. Transient transfection of fluorescent reporters represents a rapid alternative and is therefore the method of choice for analysing multiple promoters and regulatory elements. Transient transfections are also advantageous as onset rates of transcription can be measured by introducing a naked DNA template into live cells on which transcriptional complexes can assemble [12]. The latter is particularly important in cells that continuously express genes under the control of endogenous promoters. To capture the onset of expression, we must ensure all cells are labelled using an independent marker, so that cells can be tracked before expression of any fluorescent marker sets in. Identifying cells with nuclear markers, such as Hoechst, abolishes the need for co-transfection (of a second constitutively active fluorescent colour for tracking purposes), thus facilitating experiments with primary cells and comparative expression analyses of different promoter constructs. Another important aspect for our analyses is that during cell divisions the chromatin marker segregates into the two daughter cells, which aids in identifying cell divisions and assigning mother and daughter cells. Since Hoechst is excited with UV light, photodamage has to be kept to a minimum. To image over long periods of time (days) with minimal cell death, we tested UV exposure times empirically and determined 30 minute intervals to be optimal for transfected C2C12 mouse mesenchymal stem cells. During that time interval, cells exhibit significant motion, thereby greatly challenging the reliability of any tracking method.

Segmentation of nuclei is discussed in Text S1 (see also Figure S1).

### Cell tracking

Recently, software has become available for high resolution cell tracking and spatiotemporal analysis of protein dynamics in sub-cellular compartments (QuimP [13], CellTracker [14]). However, as these methods are designed to track cell boundaries in great detail, they require cells to only move by small amounts. Conventional tracking methods still require at least a minimum overlap to link cell positions between consecutive frames, measured either in absolute pixel counts, or relative to object size. This is the approach used by CellID [15], CellTracer [16], and Overlap-Based Cell Tracker [17]. If cells exhibit persistent motion and cell collisions are infrequent, ‘keyhole’ tracking algorithms can be applied, which calculate the probability of finding matching cells in a particular direction [18].

A number of single particle tracking methods have also been developed recently, which are able to track multiple non-overlapping objects and can, in principle, be applied to tracking cells [19]. Altinok et al. [20] have used spatiotemporal graph matching for tracking microtubule tips. Similarly, particle filter methods have been developed for tracking objects [6], [21], [22]. Future positions of objects are predicted using a motion model, and then matched with objects at the real positions. This usually involves solving a global linear assignment problem [23]. Both graph-based and hidden Markov model approaches can easily be extended to include additional object features, such as shape, size, colour, or texture. However, for large-scale problems, including time-series with thousands of cell positions, global optimization approaches are computationally very costly. Furthermore, particle filters only work for small displacements where motion between frames is highly correlated. In time-series with low temporal resolution and considerable cell motion, these approaches generally perform poorly.

Instead of solving a global optimization problem, we formulate here a statistical scoring approach in a less rigorous and formal way, which was briefly introduced in [24]. It is based on a similarity matrix, where scores are calculated for possible target cells within a maximum distance that can be covered by a cell in a given time interval. Relevant similarity features are selected from a larger list of possible features based on principal component analysis (PCA), similar to methods used in multi-feature cell-profiling [25], [26]. Computational demand for this local optimization problem simply scales linearly with the number of cells to be tracked.

### Constructing cell lineages

There have been some approaches to lineage construction based on the appearance or behaviour of cells during mitosis [7]. Debeir [27] computes tracking in reverse from the final frame. Divisions are detected by the merging of two daughter cells. As the cells approach mitosis, their size decreases and the two daughter cells come closer. When size and distance are below a threshold, the ‘reverse mitosis’ event has completed. Wang [28] calculates texture based features and uses feature reduction methods, including PCA to reduce 145 features to 15–20. Divisions are detected by treating each stage of the mitosis event as a hidden state in a Markov chain. A training set was used to calculate the probabilities for the chains. Similarly, Markov trees were used in [29] to map cell states to lineages.

Al-Kofahi et al. [30] construct lineages by calculating a significance score based on the observation that daughter cells have a similar size. The Ellenberg group has developed a powerful framework for automatic detection of cell divisions and chromosome phenotypes [31], [32]. Their approach, which is based on 3D time-series with stacks captured at 5–7 minute intervals, makes use of region adaptive thresholding and a feature point tracking method. Probabilities for detecting mitosis events are based on size and distance of chromosome sets for which weights are determined empirically. Li et al. [6] and a more advanced version by Bise et al. [33] use phase contrast images for cell segmentation and detection of mitosis events, which appear brighter in phase contrast. Cell trajectories are assembled into shorter fragments first, so called tracklets, which are stitched together by using a global optimisation problem a posteriori. Accuracies achieved are 87% for tracking (correctly identified cell-cell linkages between frames) and 68% for detecting divisions correctly.

Padfield et al. [34] also make use of a Hoechst label to segment nuclei, although imaging at a higher frame-rates of 6 or 15 minutes. They use a wavelet based method for cell segmentation. Subsequently, a graph flow method is used for tracking cells, and they report 99.2% of cells tracked with complete accuracy (with an average track length of 13 frames) and 97.8% correctly identified divisions, validated using 104,000 cell positions. Although the methods by Bise and Padfield are both considered state of the art, they result in markedly different detection rates and accuracies. It is difficult to pinpoint a single cause for this, but most likely it is due to experimental differences in cell density, movement and clustering. For example, the net translocation of cells observed by Padfield is small (after correction for stage drift) and thus, makes validation of large numbers of cells comparatively easy.

Comparison of different methods is almost impossible, since many of them are only available as part of an integrated commercial platform or publicly not available. Often, precision of different segmentation routines is not validated based on objective ground-truth using synthetic data, but by human observers [34], and it is difficult to obtain a comprehensive list of all parameters being used. Since there is currently no standard for exchanging track-data for evaluating different methods, we set out here to develop a new software framework using ImageJ which allows comparisons of different segmentation and tracking routines. Furthermore, we will make available validated tracked data sets at different temporal resolutions (10 and 30 min), which can be used as a benchmark test for others. The method we present here incorporates the tracking of cell lineages in our statistical scoring framework for cell tracking. It makes use of dynamic feature changes, such as characteristic changes in Hoechst distribution and nuclear size. The experimental data we make available are challenging as they are subject to considerable noise, and there is a huge variation in nuclear size and shape when compared to the examples given in Padfield [34]. Also, large cell displacements between frames make tracking by eye and validation of large numbers of cells more difficult. The clustering of cell nuclei found in our experiments poses a particular challenge when reconstructing cell lineages, as it obscures mother-daughter cell relationships.

### Current software toolkits

A software framework specifically tailored for high-throughput single cell studies is the open source image analysis platform CellProfiler [35]. CellProfiler is highly flexible and supplies all of the above mentioned segmentation methods, as well as several tracking methods including a multi-object tracker based on the method by Jaqaman [21], which accounts for splitting and merging of objects. Other tracking methods within CellProfiler utilize features such as object overlap, distance or any other measurements (intensity, morphology). A version of CellProfiler has been used for single-cell tracking by Alon et al. [3].

Here we use an alternative platform, ImageJ, which is widely used and easily extendible by Java plugins. Existing cell tracking methods for ImageJ are currently very limited, however. The Particle Tracker plugin is an implementation of Feature Point Tracking [36] and provides both segmentation and tracking based on the intensity moment of the particle images. Mtrack2 performs tracking and requires the segmentation to be performed beforehand. Trajectories are assigned by selecting the nearest particle in the following frame.

### Msx1 expression profiling

The software we developed was initially designed to measure the activity of fluorescent reporters driven by transcriptional control elements from the Msx1 gene in C2C12 mouse mesenchymal stem cells. The Msx1 protein is involved in regulating pluripotency of mesenchymal stem cells [37]. It is a member of the homeobox family of transcription factors involved in vertebrate craniofacial and muscle development. Expression of Msx1 during embryogenesis maintains progenitor cells in their undifferentiated state and mutations in the Msx1 gene lead to cranial and dental defects [38], including cleft palate. Several control elements of Msx1 have been identified by others and ourselves (Vance et al., submitted), and a key objective for the development of our analysis method was to quantify the role these elements play upon transcription rates by using fluorescent reporters. Expression levels are proportional to the amount of reporter protein provided the measured intensity is within the linear range of the imaging system. Fluorescent reporters were modified by the addition of a nuclear localization sequence (nls), which led to post-translational targeting to the nucleus. Segmentation based on Hoechst can therefore be used to measure reporter intensities in the nucleus. Ideally, we want to determine reporter levels during the lifetime of individual cells in order to avoid transgenerational inaccuracies or differences in reporter activity due to asymmetric fate choices. For this reason, methods are needed to determine reporter fluorescence between two automatically recognized cell division events in entire clonal populations.

## Materials and Methods

### Imaging of mouse C2C12 cells

C2C12 mouse myoblast cells (ECACC, Catalogue No. 91031101) were grown in DMEM supplemented with 10% foetal bovine serum at 37°C in an atmosphere of 5% CO_2_. For transient transfections, the cells were transferred to a 96-well plate at a density of 1.25×10^4^ cells per well. Hoechst 33342 (Invitrogen) 400 ng/ml in DMEM was added and incubated at 37°C for 30 minutes. Cells were then washed twice with PBS, and DMEM (without phenol red) was added. Cells in each well were subsequently transiently transfected with 200 ng of reporter plasmid using Lipofectamine 2000 (Invitrogen) according to the manufacturer's instructions.

Images were obtained using a Cellomics KineticScan KSR machine with a 10× NA 0.4 objective at a resolution of 512×512 pixels. Two colour channels (Hoechst and vGFP) were obtained every 30 minutes using the XF100 filter set. A custom import module was written to import Cellomics data (version 1.35) into ImageJ using Jackcess (version 1.1.21, http://jackcess.sourceforge.net), a library for reading and writing Microsoft Access databases.

### Imaging of zebrafish PAC2 cells

Zebrafish PAC2 cells derived from 24-hour embryos were transfected with FUCCI constructs mKO2-zCdt1(1/190)/pT2KXIGΔin and mAG-zGeminin(1/100)/pT2KXIGΔin [39], [40] and plasmid pcDNA3.1/myc-His A (Invitrogen), as previously described [41]. After neomycin selection, single cells were sorted sequentially for orange fluorescence (mKO2) and then green fluorescence (mAG) by fluorescence-activated cell sorting. A clonal FUCCI cell line was established and cultured as previously described [41]. For time-lapse analysis, FUCCI cells were plated at a density of 100,000–150,000 cells/ml onto a 35 mm glass-bottomed dish (Wilco), maintained at 28°C and imaged with a 10× NA 0.3 objective lens on an inverted Leica SPE confocal microscope. Images were captured every 15 minutes for a total of 65 hours using sequential fast scanning.

### Software design and implementation

The software was written in Java as a set of ImageJ plugins and uses the image manipulation routines available within ImageJ. The Image Viewer requires the Image5D plugin to be installed, which is available separately or bundled with the ‘Fiji’ version of ImageJ (available from http://rsb.info.nih.gov/ij/and http://pacific.mpi-cbg.de/wiki/index.php/Fiji). There are separate plugins for segmentation/tracking and viewing/editing the data.

The segmentation software can handle any image format which can be imported into ImageJ. The user selects the location to store the data and loads the image sequence into ImageJ. The segmentation parameters can be adjusted with a preview available.

The viewer allows the user to visually interact with the segmentation and tracking, and perform minor edits to the data. The application is compatible with tracking information from CellProfiler and the ImageJ plugins MTrackJ and ParticleTracker. Fluorescence time course data and cell division data can be exported as spreadsheet files. Tracking videos can be exported with highlighted cells overlaid.

## Results

**Figure 1:**
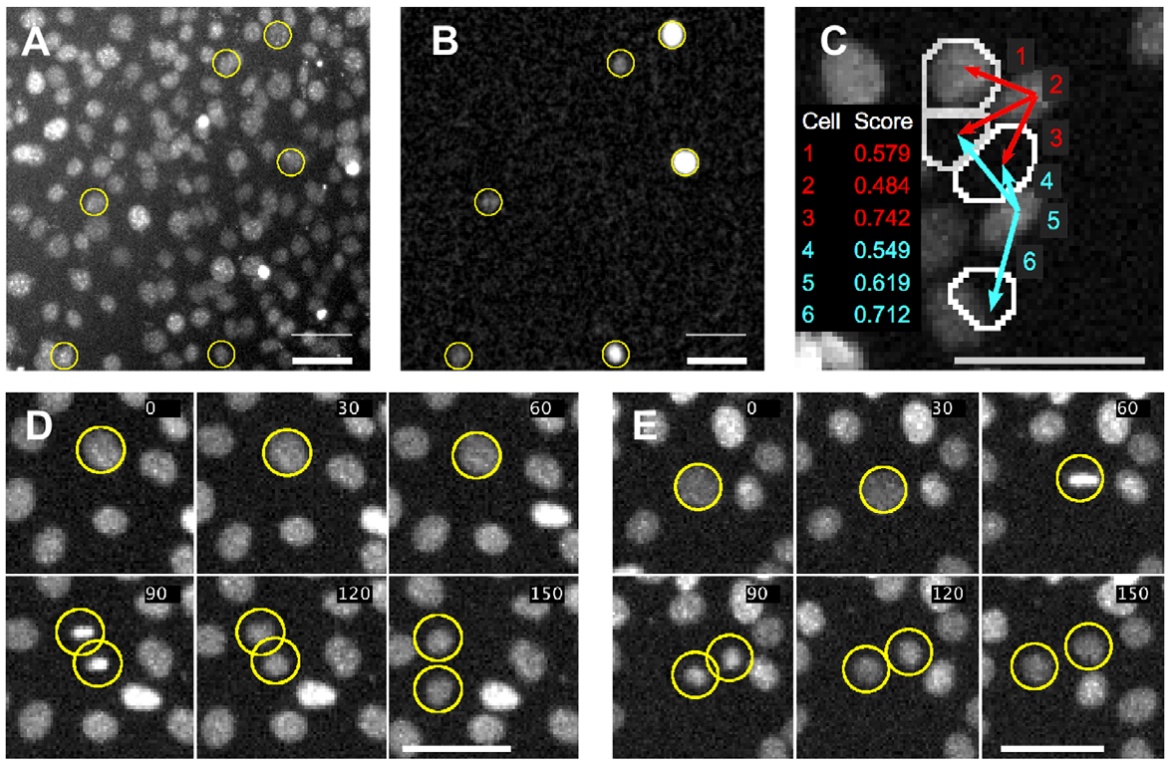
Magnified section of an image obtained from the Cellomics automated microscope. A) C2C12 cells labelled with Hoechst stain. B) Same view showing expression of GFP driven by a Msx1 promoter. GFP expressing cells have been highlighted in yellow in A and B. C) Potential ambiguity in linking cells in subsequent frames (white outlines). Arrows represent potential trajectory assignments with numbers representing the calculated score for each potential assignment. D and E) Cell divisions exhibiting chromatin condensation close to the point of division. Time is displayed in minutes. Scale bar in all images is 50 microns. (C and D have been adapted from [24], © 2011 IEEE).

Figure 1 and Figure S2 summarise the problem of tracking individual cells moving in crowded environments, and show segregation of the nuclear marker during cell divisions. Figure 1A,B show the Hoechst and GFP channels for an image with a cell density of 1300 cells/mm^2^ typically reached at t = 40 hours after transfection. The close up in Figure 1C illustrates the basic idea behind statistical scoring mechanisms for identifying matching cells in subsequent frames. For each of two example cells, three arrows point to possible target cells (white outlines) in the subsequent frame. Differently coloured arrows (e.g. red 3 and blue 4) pointing to the same target cell in the centre of the image make it obvious that positional information alone is not sufficient to discriminate which of the possible target cells is the correct one. Although connection 4 is the shortest, it turns out that connection 3 achieves the highest red score and is preferred over 4, while the highest blue score is 6. Figure 1D,E show characteristic condensation of the Hoechst marker during cell division (90 and 60 min frames), followed by segregation into daughter cells. This is an essential feature, which is used to identify cell divisions, as will be shown later on.

In the following section, we compare the efficiency and accuracy of a commercial solution, Cellomics, with different segmentation methods (for details of segmentation see Text S2). We then describe the development of the statistical scoring method for cell lineage tracking, which will be validated using a manually tracked gold standard.

### Segmentation accuracy

Two different methods were used to evaluate segmentation results, each using a different gold standard set of artificial and real cells.

Firstly, we measured the pixel-accuracy of segmentation using artificial ground truth images created by Simcep software [42]. Five frames with 2885 cell nuclei in total (at densities between 425 and 703 cells per frame to match experimentally observed cell densities) were created along with binary images, which partition the image into foreground or background. There is no additional information regarding which cell a pixel belongs to (Figure S3A, B). The F-score indicates the overall accuracy of the segmentation according to this foreground/background partitioning, but does not penalize methods which fail to separate clustered or touching cells. The precision and recall values indicate whether a segmentation method consistently over- or under-estimates the size of the detected objects. The method counts the True Positive (TP), False Positive (FP), True Negative (TN), and False Negative (FN) pixels.
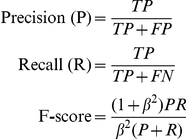



A weighting factor of *β*  = 1 was chosen to give an equal weight to precision and recall, as a combined F-score usually was found to be a good indicator of overall segmentation accuracy. The F-score performance of the different segmentation methods that have been tested is illustrated in Figure S3C. Surprisingly, the Global Threshold (Li automatic threshold from ImageJ) resulted in the highest F-values (∼0.95) for all cell densities, while the more sophisticated regional adaptive Seeded Growth and Scaling Index methods performed poorly on the artificial data (0.85<F-score<0.91).

Using the kappa index to evaluate segmentation accuracy for the Simcep data, we obtain values of KI = 0.90 (for the Seeded Growth algorithm) compared to values between 0.81 and 0.96 reported in [34]. The kappa index measures the degree of overlap between two sets:
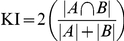



A and B are ground truth and segmented pixel data, respectively.

To demonstrate that segmentation results at higher spatial resolution are comparable to the 10× NA 0.4 images used in the rest of the paper, Figure S4 shows an image of segmented cells using a 20× NA 0.75 objective.

The second method measured positional accuracy and used images of Hoechst stained nuclei. A set of 4 frames was selected from a 48-hour period of a single experiment (frame interval 30 minutes, 110 frames in total). The images exhibited a range of cell densities from 437–730 cells per image (902–1507 cells/mm^2^); 1500 cells/mm^2^ yield 25–30% total area covered by nuclei measured using the Hoechst channel, which approximately corresponds to 90–100% cell confluency.

The nuclei were manually located using the CellCounter plugin in ImageJ. The locations as determined by regional adaptive and non-adaptive segmentation methods were then compared with these ground-truth locations. For the Seeded Growth and Scaling Index segmentation methods, we developed custom-written ImageJ plug-ins. Threshold segmentation used existing methods available in ImageJ or Fiji.

**Figure 2:**
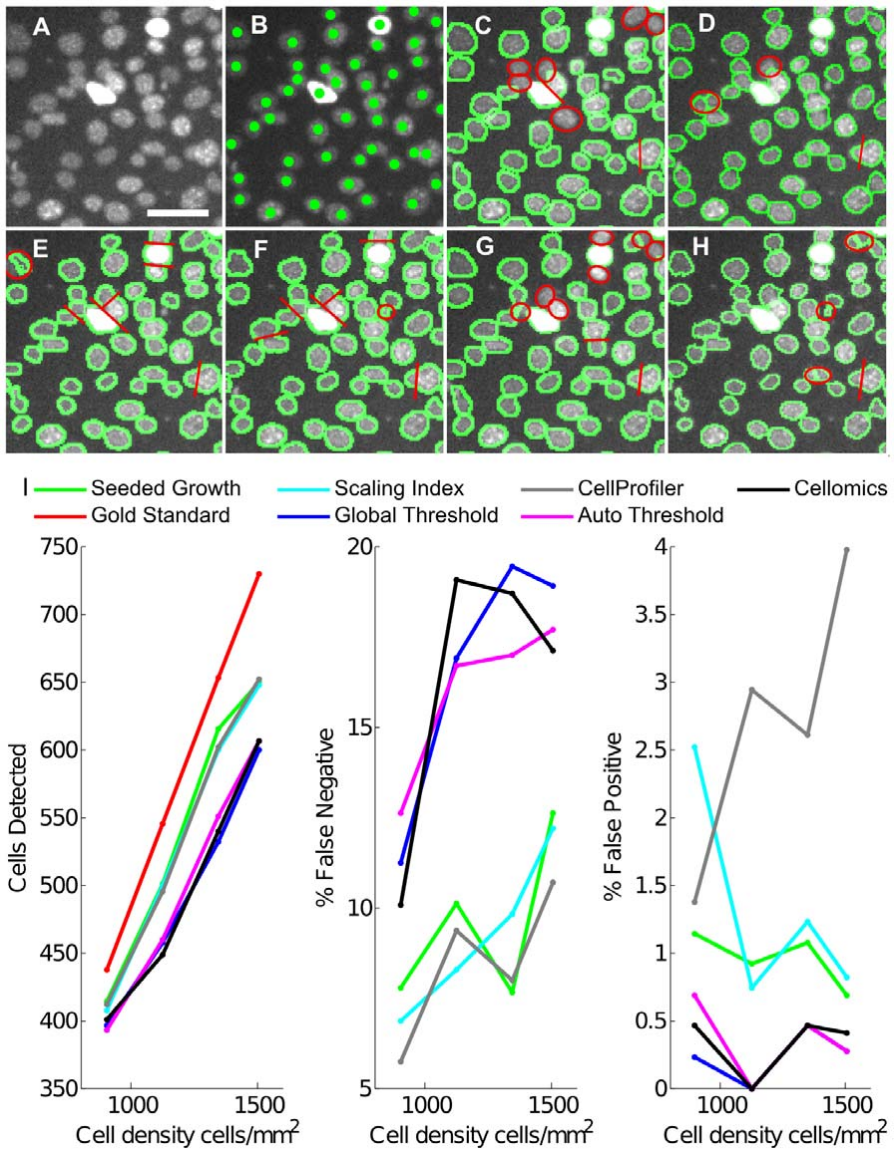
Segmentation of cell nuclei. A) Original nuclei (scale bar 50 microns) taken from the gold standard data set, cell density 1150 cells/mm^2^. B–H) Nuclei with segmentation examples overlaid. Ellipses indicate segmentation errors. Lines indicate unresolved clusters of cells. B) Manually marked cell position. C) Cellomics segmentation. D) Seeded Growth. E) Global Threshold. F) Local Threshold. G) Scaling Index. H) CellProfiler. I) Cell detection accuracy measurements: Total cell count, false negatives and false positives comparing different segmentation methods to the gold standard.

To determine positional accuracy, we define a cell as true positive when being within 1 radius of a ground-truth cell. Cells which cannot be matched are classified as false positive. Cells in the ground truth data set which remain unassigned are classified as false negative. Figures 2A–H show common problems with over- and undersegmentation encountered with different methods. Generally, it turns out that there is not a single method which outperforms all others for all cell densities (Figure 2I, and additional methods in Figure S3D), and above 1400 cells/mm^2^, detection rates decline. The Seeded Growth and Scaling Index algorithms and CellProfiler perform slightly better regarding false negatives, which are consistently below 13%. However, the simpler threshold based methods (Cellomics, Global and Auto Threshold) yield numbers of false positives (below 1%), which are well below the Scaling Index and the CellProfiler Background Adaptive method.

The large number of missed cells at high cell densities means there is currently no reliable method that can work in an unsupervised manner when cultures become confluent (in Text S3 we describe a graphical user interface for validating cell positions and eliminating falsely classified cells). We here decided to use the Seeded Growth method as it provides a good balance between false positives and negatives for different cell densities.

### Identifying features for cell tracking

During segmentation, several numerical features of nuclei are measured, similar to feature-based cell-type classification methods developed by Murphy et al. and Loo et al. [25], [26], [43], or recent methods for predicting cell fates of retinal progenitor cells using measurements of cell motion and phenotype [44].

All of the features are measured on the Hoechst nuclear channel. Additionally, the integrated intensity values are measured on the GFP channel. Our tracking algorithm combines the most informative features to compute probabilities for cell-cell transitions, which are stored in a matrix.

For the 7221 tracked positions, the measured features from Table 1 were examined using Principal Component Analysis. The first 5 principal components accounted for 74% of the variance in the Hoechst channel with the major contributions coming from mean intensity, 2^nd^ intensity moment (divided by area), nuclear area and standard deviation.

**Table 1:** Measured and derived features used in tracking.

*Feature*	*Cumulative components*			*Correlation (R^2^)*
	*1*	*2*	*3*	
**Mean Hoechst intensity** ‡	**46.95**	**97.97**	**97.97**	**0.94**
Integrated Hoechst Intensity	84.29	97.25	97.26	0.97
Median Hoechst Intensity	45.63	78.05	78.05	0.86
**Standard Deviation Hoechst intensity**	**40.64**	**91.41**	**91.42**	**0.92**
Relative standard deviation‡	5.70	58.04	58.08	0.50
2nd Intensity Moment	94.76	95.06	95.09	0.85
2nd Moment (Intensity Normalized)‡	40.86	95.82	95.91	0.78
2nd Moment (Area*Intensity Normalized)‡	47.55	91.95	92.05	0.80
**2nd Moment (Area Normalized)** ‡	**95.33**	**97.44**	**97.46**	**0.90**
**Nucleus Area**	**57.43**	**92.49**	**92.60**	**0.84**
**Integrated GFP Intensity**	**16.89**	**30.11**	**30.26**	**0.91**
Major Axis Angle	0.04	0.09	0.09	0.20
Axis Ratio	0.24	1.04	1.08	0.37
Circularity	46.95	97.97	97.97	0.16
**Centre co-ordinates of nucleus**	**N/A**	**N/A**	**N/A**	**1.00**
Δ Hoechst	6.54	9.56	9.57	0.00
Δ Area	0.22	0.23	68.22	0.01
Δ 2nd Intensity Moment	0.11	0.17	80.40	0.04
Δ Hoechst Standard Deviation	0.16	0.30	83.68	0.00
Δ Integrated GFP Intensity	0.00	0.23	0.43	0.07
Δ Circularity	0.05	0.15	43.87	0.18

Principal Component Analysis was used to determine which features contributed most to the tracking accuracy. The cumulative components columns specify how much variance of each feature is described by the first 3 principal components. Features in bold are used in the tracking system.

‡ Derived from other features. R^2^ values are given for non-dividing cells only.

**Figure 3:**
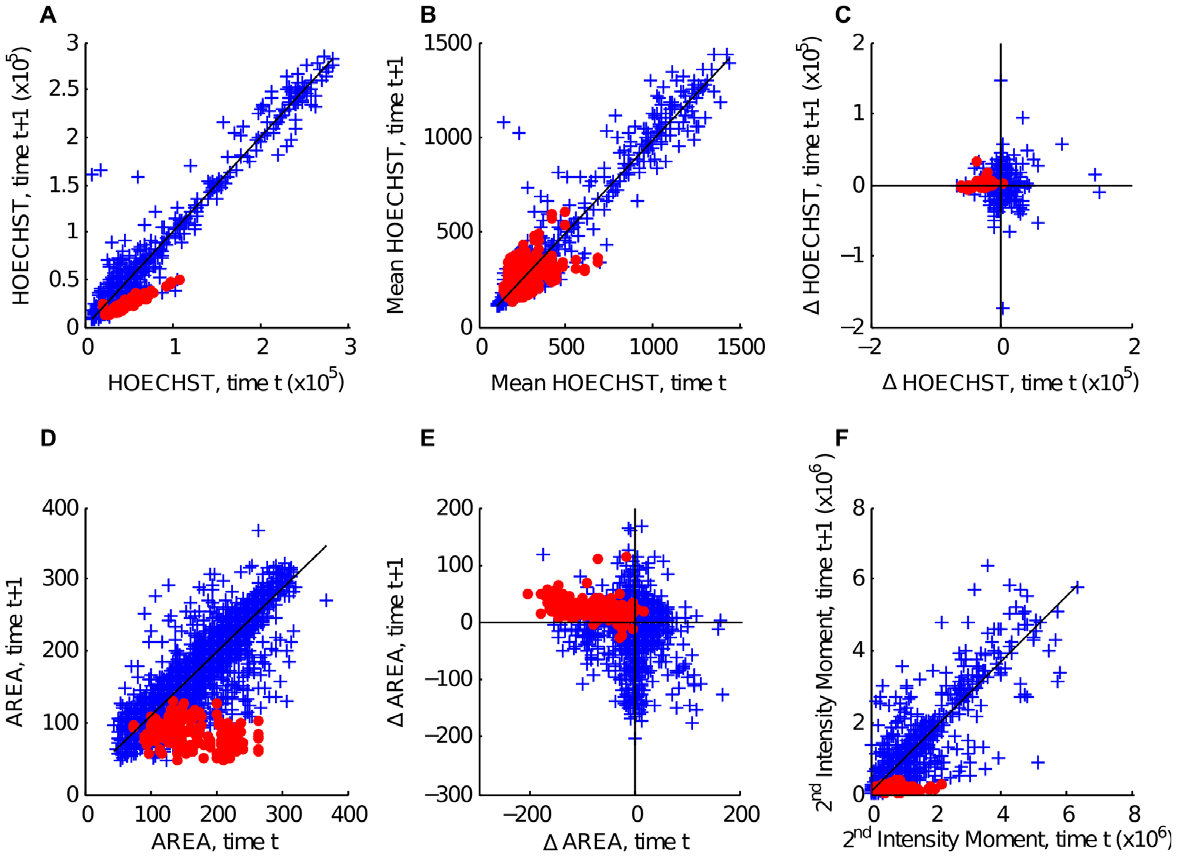
Correlations of different features between consecutive frames. Tracked cells are plotted in blue. Cells that divided between consecutive frames are plotted as red circles. R^2^ values are given only for very highly correlated values. A) Integrated Hoechst intensity. Non-dividing cells show a very high correlation in Hoechst between frames (blue R^2^ = 0.97). Red cells show that Hoechst levels are halved during division (red R^2^ = 0.90). B) Mean Hoechst intensity (blue R^2^ = 0.94). C) Change in Integrated Hoechst. D) Nucleus area. (blue R^2^ = 0.84). E) Change in nucleus area. F) 2nd Intensity moment (measured on Hoechst channel, blue R^2^ = 0.85).

The tracking algorithm relies on features remaining similar from frame to frame. Therefore, correlation scatter plots were produced, which compared the values of the features across successive frames (see Figure 3 and Figure S5). Daughter cells following division are plotted in red. For calculating correlation scores, dividing and non-dividing cells were treated separately. Dynamic features were plotted where the difference in feature value was calculated. Good features to use in tracking are ones where the values cover a wide range, while the correlation between cells in adjacent frames is good (see Table 1 for R^2^ values). According to the outcomes of principal component and correlation analysis, the following 5 features were selected for tracking: distance moved, nuclear area, mean intensity, standard deviation of intensity, 2^nd^ intensity moment (normalized to area). The feature selection was confirmed by comparing tracking accuracies for different sets of features.

### Constructing the transition matrix

Tracking is calculated on a per-frame basis with individual trajectories linking a cell in one frame with a matching cell in the next frame. For each frame, a matrix is created where the rows represent cells in the current frame and columns represent cells in the subsequent frame. Each element in the matrix holds a *movement score* representing the similarity in position and measured feature values between the cells. A value of 1 indicates that the position and feature values are unchanged between frames.

Each cell in the current frame ‘t’ is compared to the cells in the following frame ‘t+1’ and a potential trajectory is computed for each pair. Individual movement score contributions are calculated for each feature by computing the differences between the features. A threshold value determines the range over which the feature is active. 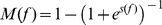
(1)where
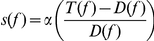



The movement score for an individual feature is given in equation (1), where *T(f)* is the threshold, *D(f)* is the difference between the values of a particular feature *f* as found in Table 1, and α determines the steepness of the curve (value to be obtained through optimization). The sigmoidal shape penalizes large changes in feature value, greater than the threshold *T*.

Threshold values are obtained by performing an initial tracking followed by analysis of the change in features (see Figure S6 and Table S1). A threshold can be selected by choosing a high percentile (95^th^–99^th^) as a cut-off, which will give a value suitable for the majority of cells in the experiment.

Each of the features has a weight which is proportional to the contribution towards the total movement score for the trajectory. Initial estimates of the weight values are obtained by determining the relative importance of each feature according to the strength of the correlation (see Figure 3, and R^2^ values in Table 1). The features with the highest correlation values (coordinates and intensity) were assigned an initial weight of 0.9 with the other features assigned weights of 0.5.

Weights and thresholds are subsequently optimized by locally varying them in an iterative manner, while maximizing the tracking performance. Each parameter is perturbed in turn by a small amount (±1% of the parameter range) with the new values retained if the tracking score is improved. The optimizer attempts to avoid local minima by gradually increasing the scale of the perturbations if repeated iterations fail to improve the score.

The individual scores are combined using equation (2) as the product of all feature weights and movement scores.
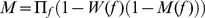
(2)


### Assigning trajectories

Assigning movements is a four-stage process (see Figure S7). The first step builds a list of potential target cells in the adjacent frames according to the movement scores in the transition matrix. Each cell holds a list of highest scoring cells in both the forward (t→t+1) and backward (t→t−1) directions.

The second stage assigns a trajectory if the highest scoring forward transition agrees with the highest scoring inbound transition of the target cell at t+1 (see Figure S8). Step 2 is performed repeatedly until all such transitions have been assigned. The third step completes any remaining links by assigning the highest forward pointing transition.

The final step optimizes the tracking by calculating the sum of transition scores for each frame. If two cells share potential targets, a new transition score is calculated based on exchanging the trajectories. The new trajectories are retained if the exchange improves the total score.

The method of assigning trajectories may be replaced with the Hungarian Algorithm [45], [46], while retaining the initial matrix calculation. The Hungarian method requires a square matrix; therefore an additional step is required to pad the matrix where there are different numbers of cells in adjacent frames. Although the tracking accuracies with the Hungarian method are very similar, the main advantage of our custom assignment is that it is capable to account for the detection of cell divisions.

### Detection of divisions

The large frame intervals used in the C2C12 experiments lead to difficulties in identifying cell divisions. The M-phase of the cell cycle is relatively brief and can occur between frames; therefore, the change in appearance of the nucleus during M-phase cannot be relied upon to detect divisions. Also, directional information about daughter cells moving in opposite directions during division could not be used, as there was no significant correlation observed between frames.

The first step in locating divisions is to identify cells which may have divided by making use of dynamic features obtained during tracking, in particular, characteristic changes in intensity and nuclear area (Figure 3 C,E), which both decrease by at least 25% during cell division (Figures S9 and S10).

The integrated intensity of the parent cell is very closely retained in the daughter cells (R^2^ = 0.95, sum of daughter intensities is 100±1.5% of parent cells, errors indicate standard error of the mean, n = 100 cell divisions), and there is a close correlation between the two daughter cells (R^2^ = 0.92, mean difference between daughter cells 6.0±0.5%). The daughter cells in the frame immediately following a division were of a similar size to each other (average difference 12.6±1.0%), and for the sum of daughter cell areas we obtain an average total 110±4.3% of parent cell area. There were some cases where a daughter cell was larger than the final measured area of the parent cell due to the long frame interval and chromatin condensation occurring during the previous frame. Because of this and the larger variation obtained for the area, cell size (weight 0.25) is weighted lower than intensity (weight 1).

Potential daughter cells are selected by examining cells within a certain distance of the parent cell. These cells are examined one pair at a time, and a similarity score is calculated using equation (2) based on intensity and size only. The most favourable daughter pairs are compared to the parent cell by re-evaluating equation (2) using a ‘composite cell’ where the area and intensities are the sums of the daughter values, again using weights of 1 and 0.25 for intensity and area, respectively. Finally, daughter cells with the highest score are selected.

### Tracking accuracy

**Figure 4:**
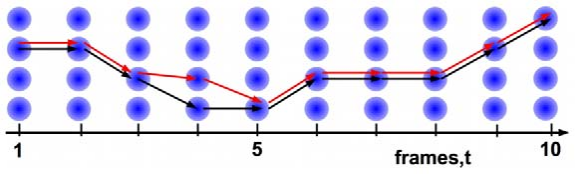
Measuring tracking accuracy. Horizontal axis shows time with the vertical axis representing cells in the frames. The red line is the manually tracked ‘gold standard’ route marked through the cells, and the black line is the calculated tracking. Tracking accuracy is measured by counting the total number of steps which match the gold standard and the longest continuous chain of correct steps.

To compare tracking accuracies of our method with CellProfiler and ImageJ's Particle Tracker (https://weeman.inf.ethz.ch/ParticleTracker), we used an experiment with 24 frames in total (frame intervals of 10 minutes). The average cell movement between frames was 3.9 pixels, with a maximum of 28 pixels (average nucleus diameter was 11 pixels). The cell density (1300 cells/mm^2^) was in the middle of the range of our 30 minute experiment described earlier. We created a gold standard, whereby the segmentation and tracking were manually adjusted until at least 50% of the visible cell nuclei had been tracked. The gold standard contains 7017 individual cell to cell linkages between frames, with 359 tracks ranging from 5 to 23 frames (average 19). The tracking accuracy was measured by counting the number of individual links that were correctly identified using the automated methods and the longest continuously tracked section (Table 2, Figure 4).

**Table 2:** Results of gold standard tracked sets.

*Experiment:*	*24 frames (10 minute interval), gold standard.*	*110 frames (30 minute interval), gold standard.*
Validated Positions	7321	7417
Validated Trajectories	359	157
Frame to Frame links	6886	7221
Average track length	19	46
*Tracking Scores:*		
LineageTracker (Custom assignment)	97.7/91.8	97.2/85.3
LineageTracker, (Hungarian Assignment)	98.1/94.2	96.9/89.1
CellProfiler*	95.9/88.3	96.1/85.4
Particle Tracker (ImageJ)	92.3/82.9	86.4/64.1
Cellomics	n/a	85.9/55.9

Two numbers are given for each measurement: total number of correctly tracked steps and longest continuously tracked section (as percentage of total steps). For the 10 minute interval experiment, the seeded growth algorithm was used, and segmentations were manually edited, so that 50% of cells with positively validated segmentations were included in the tracking gold standard. The 30 minute interval experiment is based on the Cellomics segmentation, as to allow comparison of the Cellomics tracking routines with other ones.

*CellProfiler tracking using LAP (Linear Assignment Problem) tracking.

While our custom method with 97.7% correctly identified linkages compares similarly to CellProfiler (95.9%), ImageJ's Particle Tracker more generic feature point tracking, which like our method also includes intensity and higher order intensity moments as features, has a slightly lower detection rate of 92.3%. Next, a tracking ‘gold standard’ was created using the longer 48 hour time-series data with 30 minute frame intervals from the same experiment used for the segmentation standard. 157 cell trajectories were created in our tracking viewer/editor containing a total of 7221 individual steps. Track lengths range from 5 to 110 frames (average 46). Average cell movement was 3.8 pixels per frame (maximum 29 pixels per frame, average cell diameter of 14 pixels). Additionally, this experiment includes 100 cell divisions. Results for our method and CellProfiler are very similar to the previous experiment, whereas the Particle Tracker plugin shows a markedly decreased rate of accuracy for the longest continuously tracked section (Table 2), possibly because of higher cell densities encountered in the 30 min interval experiment.

Execution times are comparable for all methods, taking approximately 1–2½ minutes on a 2.4GHz Intel Core i5 running OSX 10.6.7. These times decrease for the custom tracking when an optimized value for the Distance Threshold is used, to below 10 seconds for the custom assignment and approximately 1 minute for the Hungarian assignment.

### Division accuracy and daughter cell fluorescence

**Figure 5:**
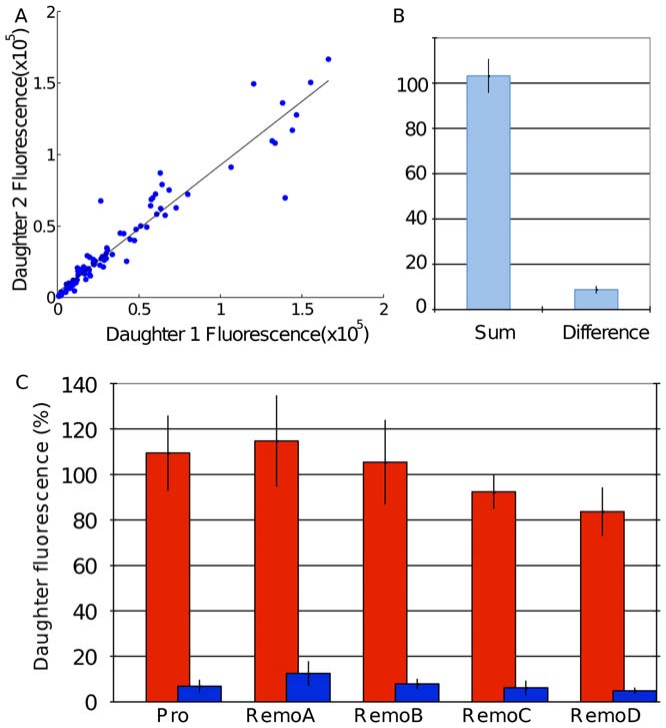
GFP Fluorescence measurements across cell divisions. A) Correlation plots of daughter fluorescence (R^2^ = 0.92) taken from the 5 Msx1 ReMo constructs. B) Sum of daughter fluorescence and difference between daughter fluorescence, as a percentage of parent fluorescence. C) Breakdown of sum and difference of intensities for the 5 different Msx1 ReMo constructs.

The main purpose of our software development was to create a framework that allowed tracking of cells through cell divisions. To determine the accuracy of detecting cell divisions, we considered the 110-frame experiment. Out of the 100 manually annotated cell divisions, 80 were correctly identified by the software. There were 16 false positive divisions detected: two where a division was correctly identified, but the daughter cells were assigned incorrectly, and the remaining 14 where a division was detected and none occurred. In a series of additional experiments, our software was used to study the partitioning of a cis-regulatory module promoter driven GFP between daughter cells for dividing C2C12 cells. Transient transfections were performed with reporters containing four different Msx1 transcriptional regulatory regions (A–D) upstream of the Msx1 promoter and the promoter alone (Vance et al., submitted). The fluorescence activity of mother and daughter cells was measured for a total 96 divisions. These cells were manually validated. The partitioning between daughters is summarized in figure 5A (R^2^ = 0.92). The high correlation in the partitioning means that for all the different Msx1 promoter constructs driving GFP expression, we find that fluorescence is symmetrically distributed in the two daughter cells with a high degree of accuracy, ensuring that in most cases Msx1 levels are maintained during cell divisions to prevent differentiation. The total fluorescence recovery (measured as the percentage of fluorescence in the daughter cells compared to the mother cell) is summarized in figure 5B, C. A correlation between mother fluorescence and total daughter fluorescence yields an R^2^ value of 0.86. This lower value most likely reflects degradation of GFP during cell division, when transcription of GFP under the control of the Msx1 promoter ceases.

### Tracking cells without a permanent nuclear marker

**Figure 6:**
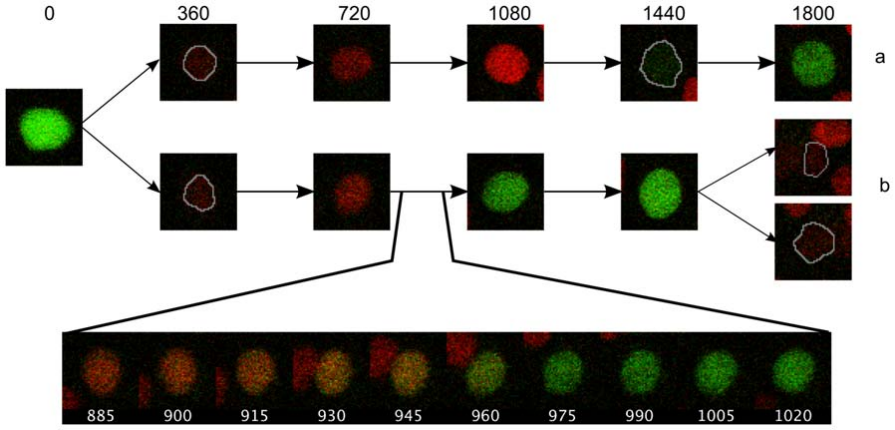
Colour changes during the cell cycle indicated by FUCCI markers in two daughter cells labelled a and b (see also **Figure 7**). Time is in minutes following division. The overlap in the red and green fluorescence (transition between G1 and S phase) is shown for cell b (bottom panel). White outlines are given for nuclei showing weak fluorescence.

**Figure 7:**
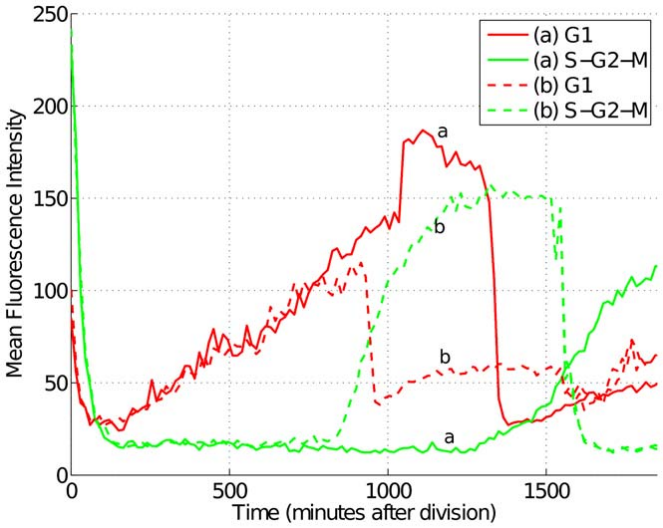
Intensities of the FUCCI markers following cell division. Fluorescence intensity following cell division for the two daughter cells in figure 6. The two FUCCI channels have been shown for an entire cell cycle. The G1 signal (red) increases gradually following mitosis, then decreases following a rise in S-G2-M signal (green). A magnified view of the first 3 hours is shown in Figures S11 and S12.

The software was originally designed to track cells which contained a continuously visible fluorescent marker. To show that this is not an absolute requirement, we use it here to obtain intensity profiles of zebrafish embryonic PAC2 cells, expressing FUCCI cell cycle markers visible for the most of the duration of the cell cycle. The markers consist of two ubiquitin ligase substrates, which are expressed during different phases of the cell cycle [39] and have been fused with red- and green-emitting fluorescent proteins [40]. The nuclei of cells in the G1 phase appear red and change to green during the S, G2 and M phases of the cell cycle (Figures 6 and 7). There is an overlap during the G1 to S transition where both markers are visible, giving the nuclei a yellow colour (Figure 6, bottom panel). At mitosis, there is a rapid decrease in intensity in the green channel, but there is a short delay before the cell becomes visible in the red channel. Because of that delay, there is insufficient difference between daughter cells and background for accurate automatic detection, so manual intervention is required for a short section of each lineage (Figures S11 and S12 and Table S2). As described in , differences in the colour channels inform the seeded growth algorithm, as well as the tracking module in order to facilitate discrimination between nearby cells at different phases of cell cycle (see also Figure S13).

### Conclusions

Currently, there are few alternatives for automated cell tracking that are freely available, such as CellTracker, CellID, CellProfiler, CellTracer, and Overlap-Based Cell Tracker. All of them have shortcomings with large cell displacements between frames, and do not allow for automated cell lineage construction. Our method, which is based on the ImageJ plugin architecture, has demonstrated a similar performance to CellProfiler when it comes to cell segmentation, but has the added feature of cell lineage construction capabilities, and the advantage to interactively correct segmentation or tracking mistakes.

It can read data files produced from CellProfiler to allow visualization and editing of segmentation and tracking output, in order to compare between different tracking solutions implemented in CellProfiler and ImageJ. The Seeded Growth segmentation we used detected cells with 92% accuracy with <1% false positives. Cell tracking followed entire trajectories (of mean length 45 cell-cell transitions) with 85% accuracy. This is similar to results in [33], but does not reach the higher accuracies reported in [34], in which cells exhibit less motion between frames and are less clustered. The gold standard we release (15,000 validated cell positions) has a longer average of 19 and 46 tracked frames for the 10 min and 30 min interval experiments with 359 and 157 tracks for each of the experiments when compared to an average track length of 13 frames in [34]. We found for different Msx1 promoter constructs that there is a high level of accuracy when distributing GFP fluorescence to daughter cells during cell divisions. Additionally, as shown in the example of FUCCI cell cycle markers, our software can be easily adapted to different cell types and fluorescent markers.

### Availability and future directions

The software and source code can be downloaded from http://go.warwick.ac.uk/lineagetracker. Additional segmentation or tracking methods are possible by adding modules for tracking or lineage construction within the software. Current segmentation methods have been optimized for circular nuclei. Different methods could be substituted for segmenting different shapes, such as rod-shaped yeast or bacterial cells, or when using different fluorescent stains, such as GFP-histone for labelling cell nuclei [47].

The tracking comparison and benchmarking software will be made available from the lineagetracker website.

Our statistical scoring framework can, in principle, be translated into a more formal framework of a graph based problem, as used by Padfield [34] or others. Here we have chosen it for the simplicity with which it can be implemented and the ease in which dynamic features can be incorporated.

## Supporting Information

Figure S1 **Distribution of nuclei sizes follows a gamma distribution.** A) 110 frames (30 min intervals) experiment of C2C12 cells (n = 62586 , γ = 7.4 , β = 20.2). B) Analysis of the first three frames of the sequence showing the distribution of all nuclei that have been automatically identified using the built-in Cellomics segmentation (1235 cells, blue and red), Blue is a subset of nuclei that have been manually validated to be non-overlapping (n = 1198). The corresponding gamma curve has parameters γ = 11.1 and β = 12.0. Red contains nuclei that have been confirmed to be overlapping by visual inspection (35 nuclei, 2.8% of total), i.e. where two nuclei were reported as one. 1 nucleus was oversegmented, i.e. falsely reported as two.
(TIF)

Figure S2 **Example of C2C12 cell motion.** The highlighted cell has been tracked through multiple frames. Scale bar is 50 microns. Time is displayed in minutes. A) Hoechst channel B) GFP Channel.
(TIF)

Figure S3 **Segmentation score plots.** A) Artificial cell images from Simcep [42]. B) Ground Truth image. C) Precision, Recall & F-Score for the SimCep images. D) Comparison of cell detection accuracies for various segmentation methods.
(TIF)

Figure S4 **Segmentation of C2C12 cells at a higher resolution, obtained using a 20× NA 0.75 objective.**
(TIFF)

Figure S5 **Correlation plots with dividing cells coloured in red.** Top: Change in Hoechst intensity, Change in 2nd order intensity moment, Correlation in standard deviation. Bottom: intensity correlations for daughter cells, parent fluorescence against sum of daughter fluorescence, parent cell area against sum of daughter areas.
(TIF)

Figure S6 **Measuring changes in features for cell-cell transitions during tracking.** A) Change in cell areas (pixels) in adjacent frames. B) Distance moved by non-dividing cells in one frame. C) Percent change in Hoechst fluorescence for non-dividing cells. D) Distribution of daughter cell distances (in pixels) from parent cell in the frame immediately following a division.
(TIF)

Figure S7 A) Tracking flow chart. B) Expanded flow chart for the Detect Divisions module. (Adapted from [24] © 2011 IEEE).
(TIF)

Figure S8 **Demonstration of three iterations of the assignment step.** 1, 2 & 3 represent three cells in time t, a, b & c are three cells at time t+1. Numbers on arrows indicate movement scores. A) The highest scoring link between 2-c is selected. B) Links to and from cells 2 & c are removed. The highest scoring link 3-b is selected. C) Links involving cells 3 & b are removed, leaving 1-a.
(TIF)

Figure S9 **The cell divisions from** **figure 1B** **, showing changes in Hoechst intensity.** For each row, the left plot displays the integrated Hoechst intensity; the right plot displays mean Hoechst intensity. (S9A adapted from [24] © 2011 IEEE).
(TIF)

Figure S10 **Cell tracked across 3 generations.** A) Intensity profile of the lineage showing GFP fluorescence. B&C) Highlighted sections of the cell trajectory. Tracks are colour coded to match the intensity plot. Inset shows the cell highlighted.
(TIF)

Figure S11 **Intensity drop following division for zebrafish PAC2 cells.** The image background intensity and sum of image channels for the measured cell are also plotted.
(TIF)

Figure S12 **Dividing cell visualised using FUCCI markers.** The green FUCCI S-G2-M marker fades after mitosis followed by a slow increase in red G1 marker. Time displayed in minutes same as Figure S11 above.
(TIF)

Figure S13 **Segmentation of zebrafish PAC2 cells using the ‘Multi-Channel Segmentation’ method.**
(TIF)

Table S1 90–99th percentile values for change in area, frame to frame displacement during tracking, and parent-daughter distance following cell division. These values (measured in pixels) are used to select the initial threshold parameters used for tracking.
(PDF)

Table S2 Tracking precision for zebrafish PAC2 cells visualised using FUCCI markers [39]–[41]. The segmentation and tracking adjustments represent the percentage of frames which required manual intervention to preserve accurate tracking. The longest continuous sequence was observed with cell 8 at over 50 hours without corrections. Following division, daughter cells fade to close to background intensity requiring cells to be manually segmented.
(PDF)

Text S1 **Segmentation of cell nuclei.**
(PDF)

Text S2 **Description of algorithms and parameters used for segmentation.**
(PDF)

Text S3 **Description of LineageTracker software user interface.**
(PDF)
