# Supplementary figures and images for: Extracting Fluorescent Reporter Time Courses of Cell Lineages from High-Throughput Microscopy at Low Temporal Resolution
Source: PLoS One. 2011 Dec 15;6(12):e27886. doi: 10.1371/journal.pone.0027886 (PMC3240619; doi:10.1371/journal.pone.0027886)

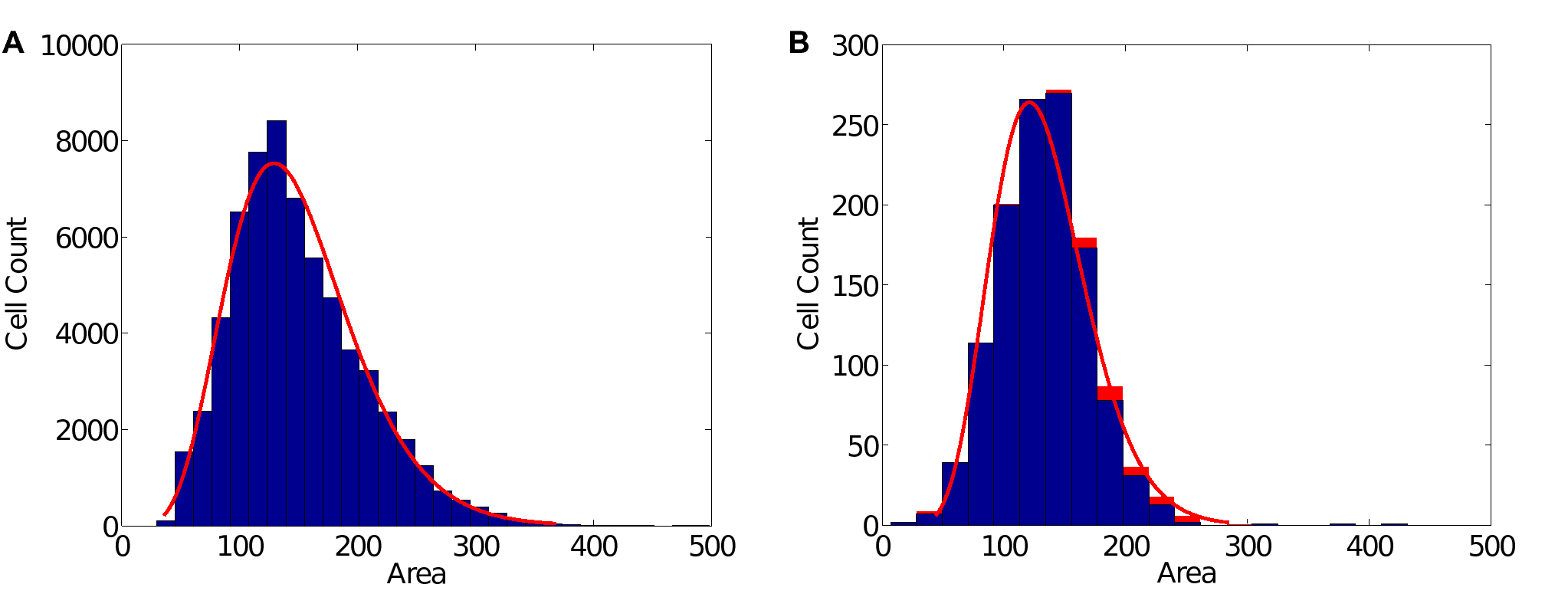

Supplement: Figure S1 — Distribution of nuclei sizes follows a gamma distribution. A) 110 frames (30 min intervals) experiment of C2C12 cells (n = 62586 , γ = 7.4 , β = 20.2). B) Analysis of the first three frames of the sequence showing the distribution of all nuclei that have been automatically identified using the built-in Cellomics segmentation (1235 cells, blue and red), Blue is a subset of nuclei that have been manually validated to be non-overlapping (n = 1198). The corresponding gamma curve has parameters γ = 11.1 and β = 12.0. Red contains nuclei that have been confirmed to be overlapping by visual inspection (35 nuclei, 2.8% of total), i.e. where two nuclei were reported as one. 1 nucleus was oversegmented, i.e. falsely reported as two. (TIF) [file pone.0027886.s001.tif]

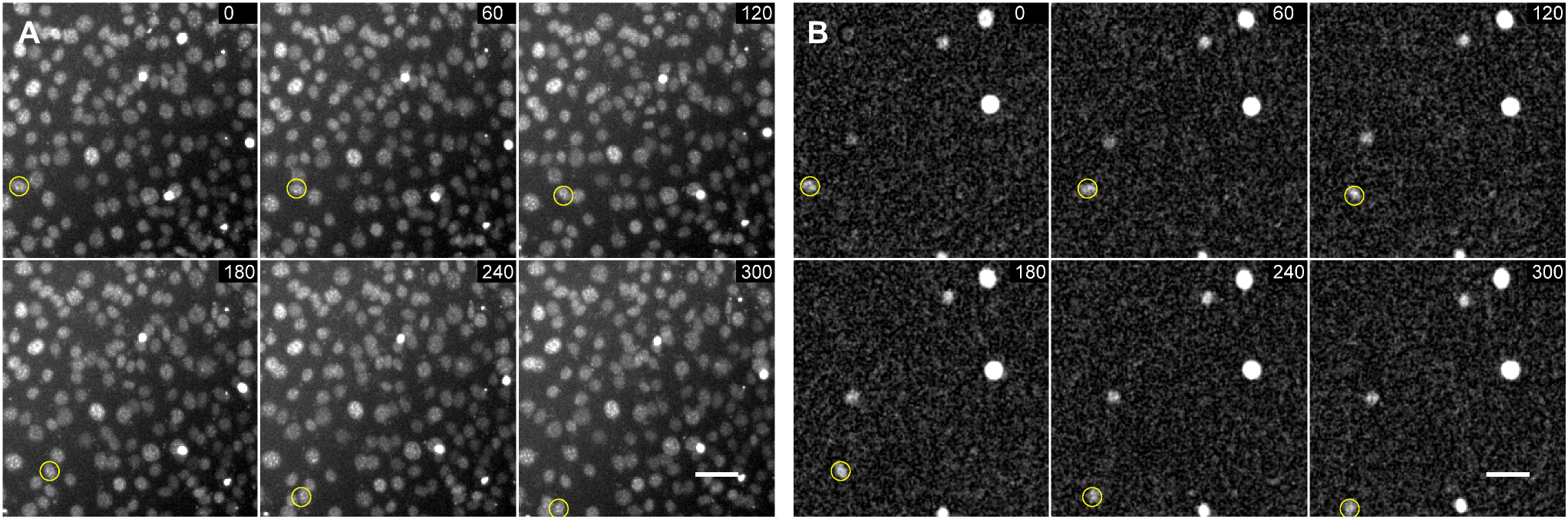

Supplement: Figure S2 — Example of C2C12 cell motion. The highlighted cell has been tracked through multiple frames. Scale bar is 50 microns. Time is displayed in minutes. A) Hoechst channel B) GFP Channel. (TIF) [file pone.0027886.s002.tif]

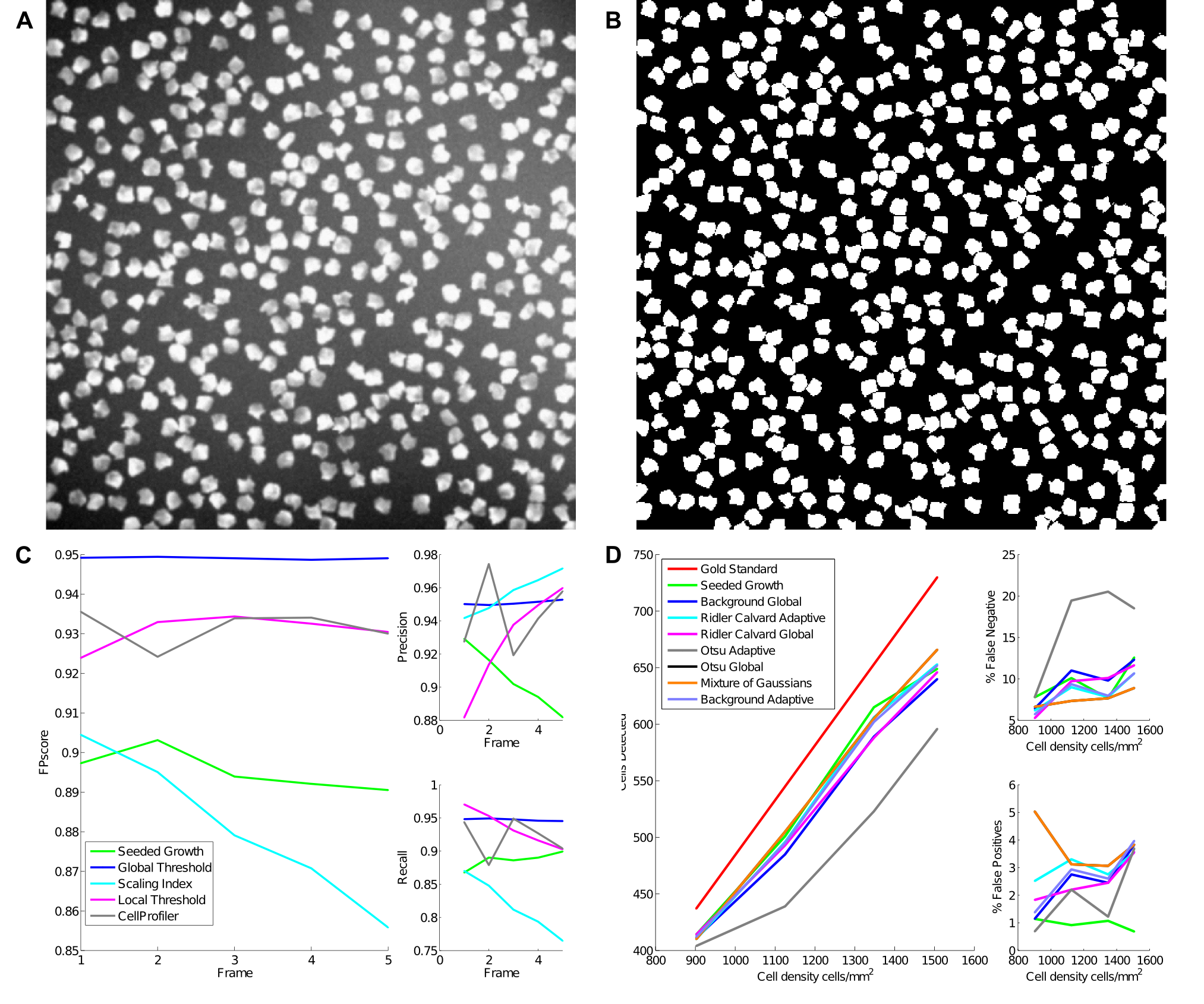

Supplement: Figure S3 — Segmentation score plots. A) Artificial cell images from Simcep [42]. B) Ground Truth image. C) Precision, Recall & F-Score for the SimCep images. D) Comparison of cell detection accuracies for various segmentation methods. (TIF) [file pone.0027886.s003.tif]

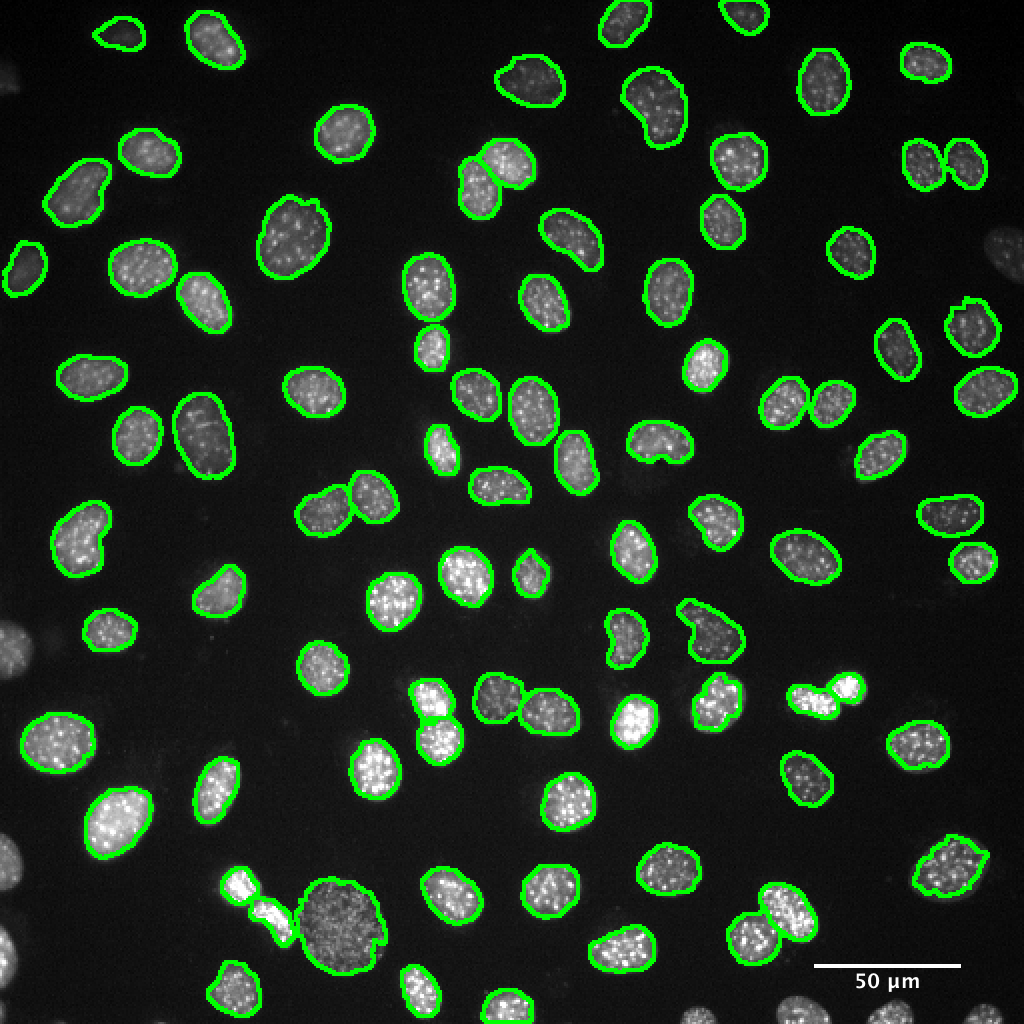

Supplement: Figure S4 — Segmentation of C2C12 cells at a higher resolution, obtained using a 20× NA 0.75 objective. (TIFF) [file pone.0027886.s004.tif]

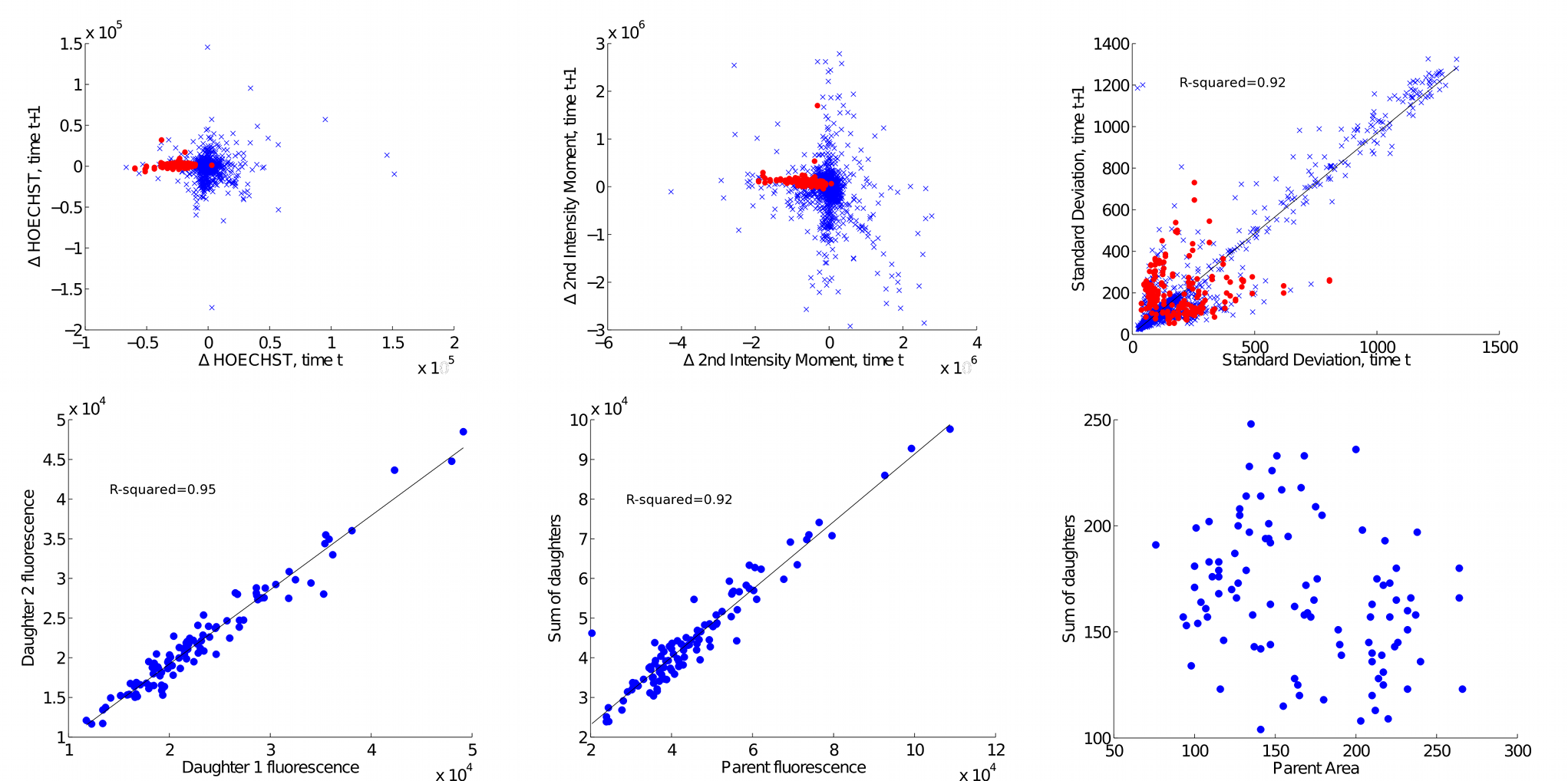

Supplement: Figure S5 — Correlation plots with dividing cells coloured in red. Top: Change in Hoechst intensity, Change in 2nd order intensity moment, Correlation in standard deviation. Bottom: intensity correlations for daughter cells, parent fluorescence against sum of daughter fluorescence, parent cell area against sum of daughter areas. (TIF) [file pone.0027886.s005.tif]

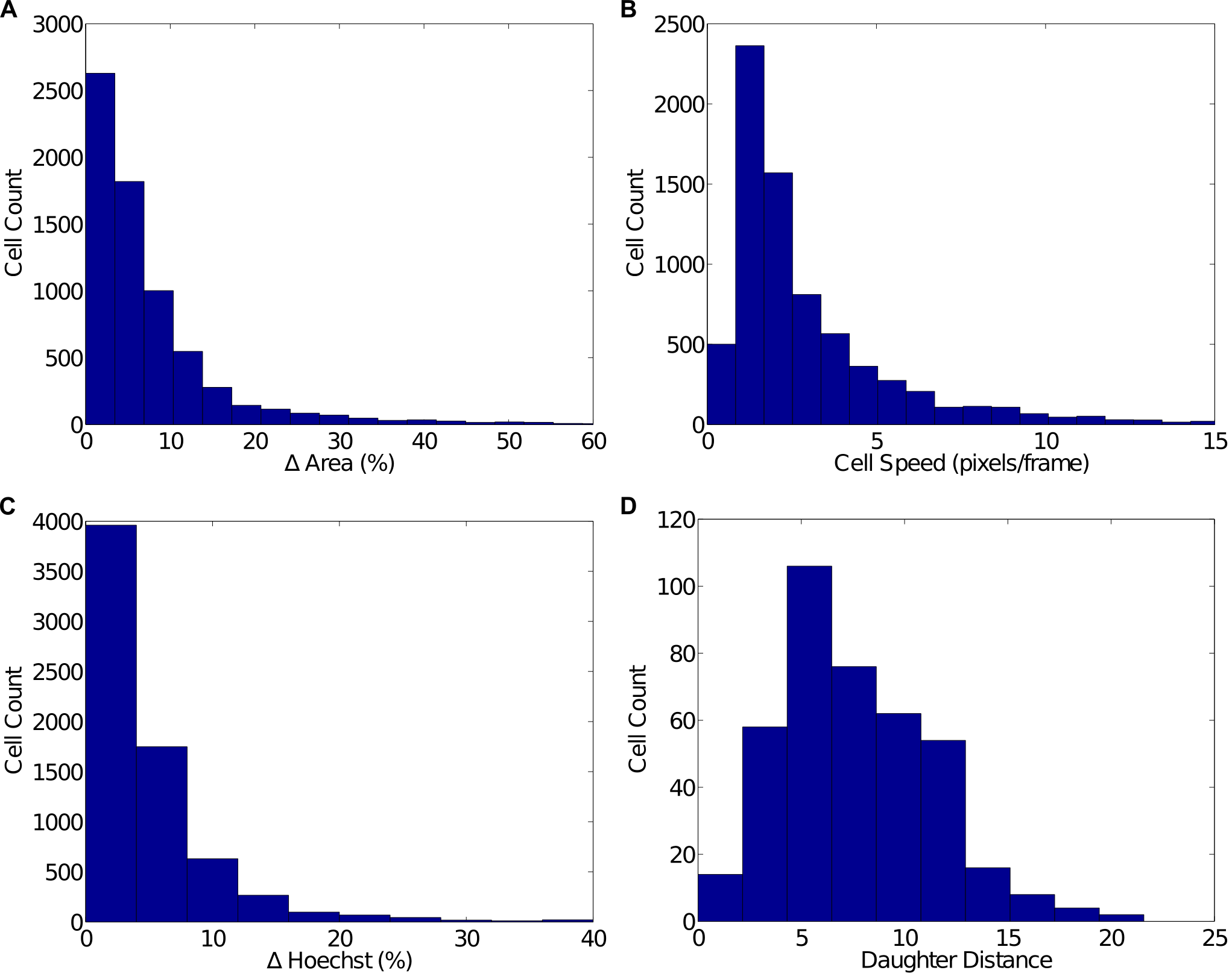

Supplement: Figure S6 — Measuring changes in features for cell-cell transitions during tracking. A) Change in cell areas (pixels) in adjacent frames. B) Distance moved by non-dividing cells in one frame. C) Percent change in Hoechst fluorescence for non-dividing cells. D) Distribution of daughter cell distances (in pixels) from parent cell in the frame immediately following a division. (TIF) [file pone.0027886.s006.tif]

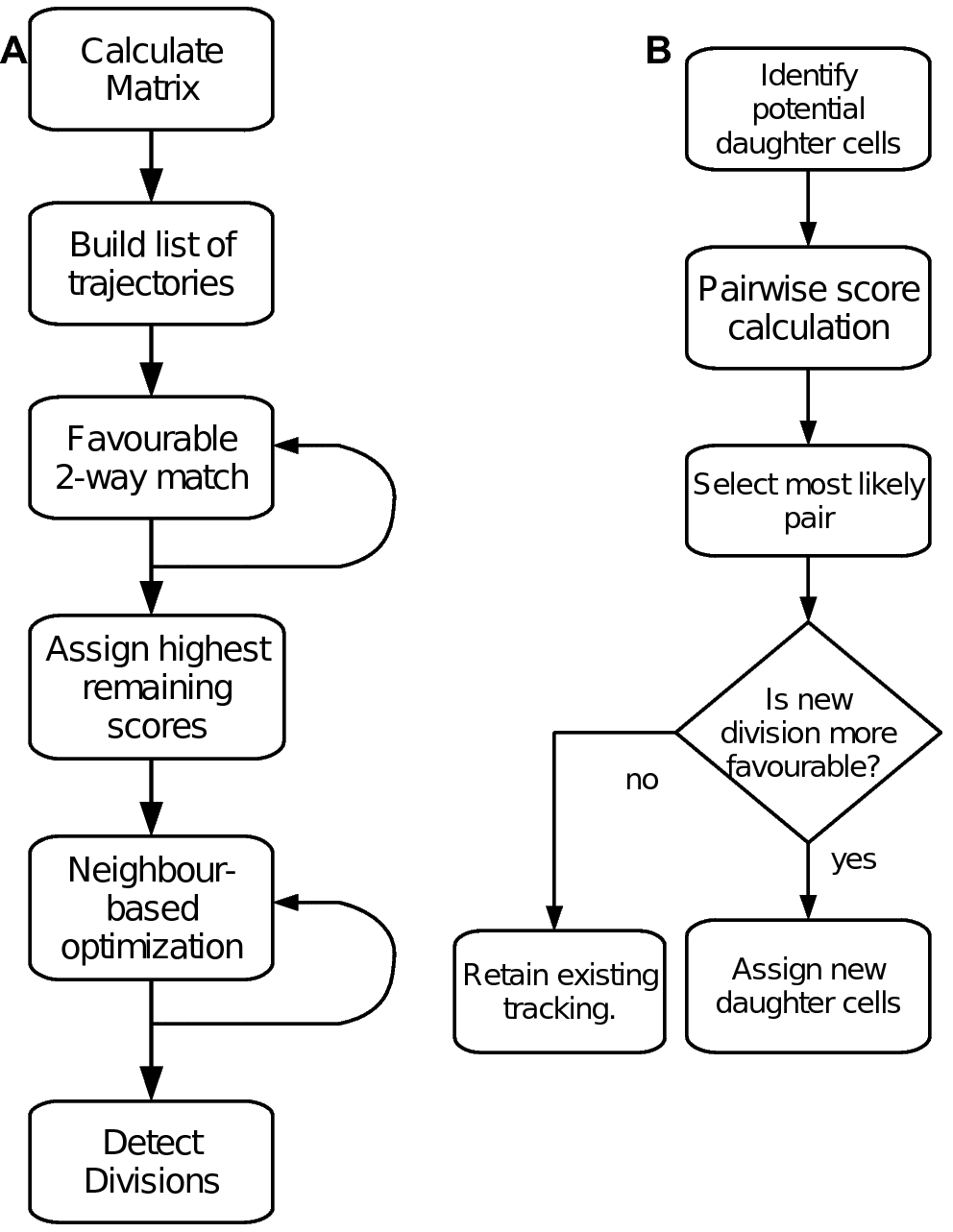

Supplement: Figure S7 — A) Tracking flow chart. B) Expanded flow chart for the Detect Divisions module. (Adapted from [24] © 2011 IEEE). (TIF) [file pone.0027886.s007.tif]

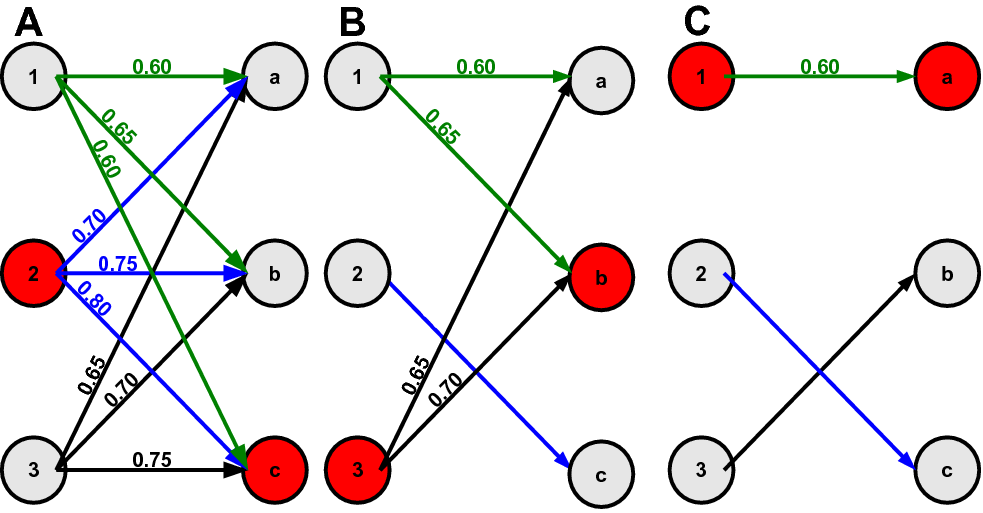

Supplement: Figure S8 — Demonstration of three iterations of the assignment step. 1, 2 & 3 represent three cells in time t, a, b & c are three cells at time t+1. Numbers on arrows indicate movement scores. A) The highest scoring link between 2-c is selected. B) Links to and from cells 2 & c are removed. The highest scoring link 3-b is selected. C) Links involving cells 3 & b are removed, leaving 1-a. (TIF) [file pone.0027886.s008.tif]

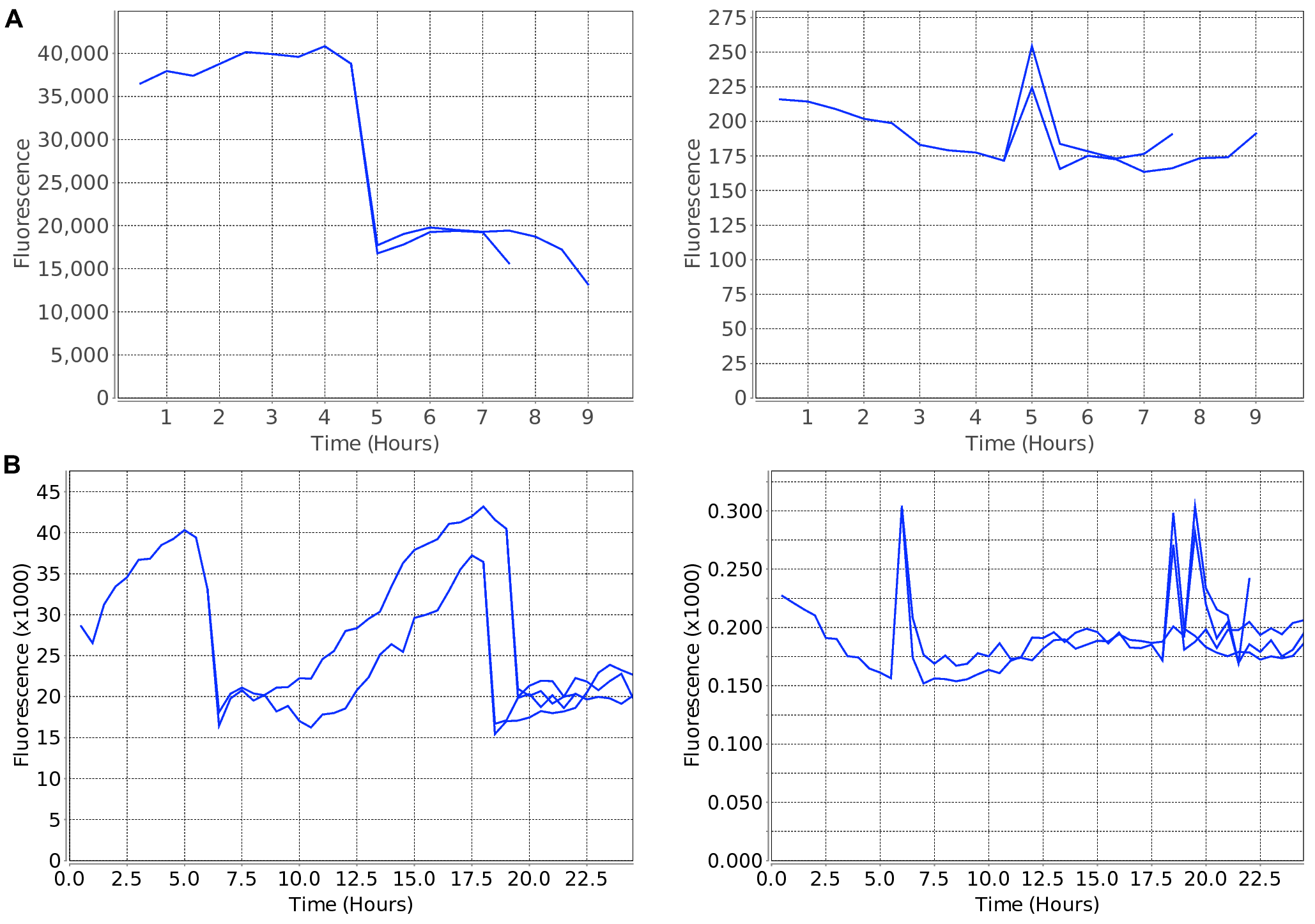

Supplement: Figure S9 — The cell divisions from figure 1B , showing changes in Hoechst intensity. For each row, the left plot displays the integrated Hoechst intensity; the right plot displays mean Hoechst intensity. (S9A adapted from [24] © 2011 IEEE). (TIF) [file pone.0027886.s009.tif]

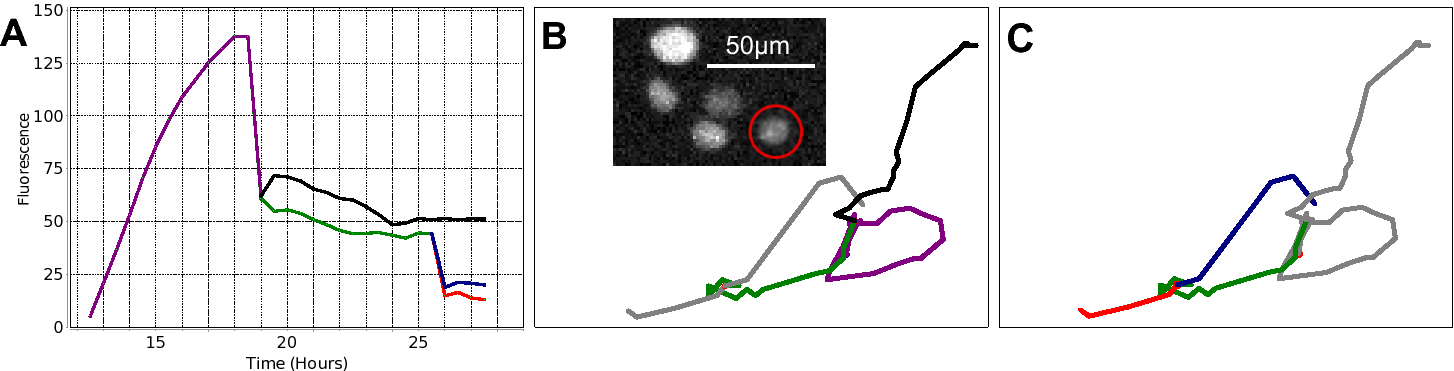

Supplement: Figure S10 — Cell tracked across 3 generations. A) Intensity profile of the lineage showing GFP fluorescence. B&C) Highlighted sections of the cell trajectory. Tracks are colour coded to match the intensity plot. Inset shows the cell highlighted. (TIF) [file pone.0027886.s010.tif]

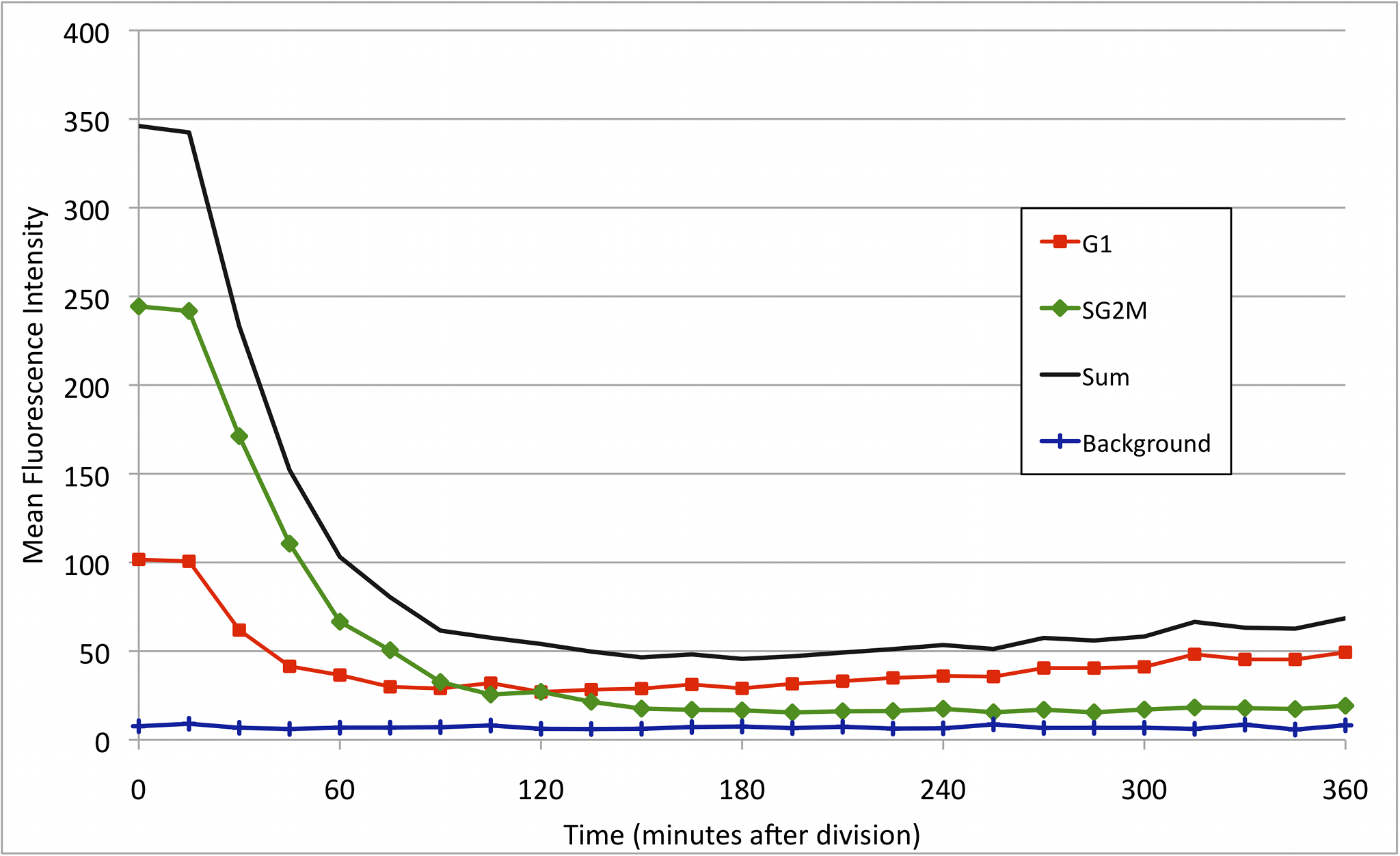

Supplement: Figure S11 — Intensity drop following division for zebrafish PAC2 cells. The image background intensity and sum of image channels for the measured cell are also plotted. (TIF) [file pone.0027886.s011.tif]

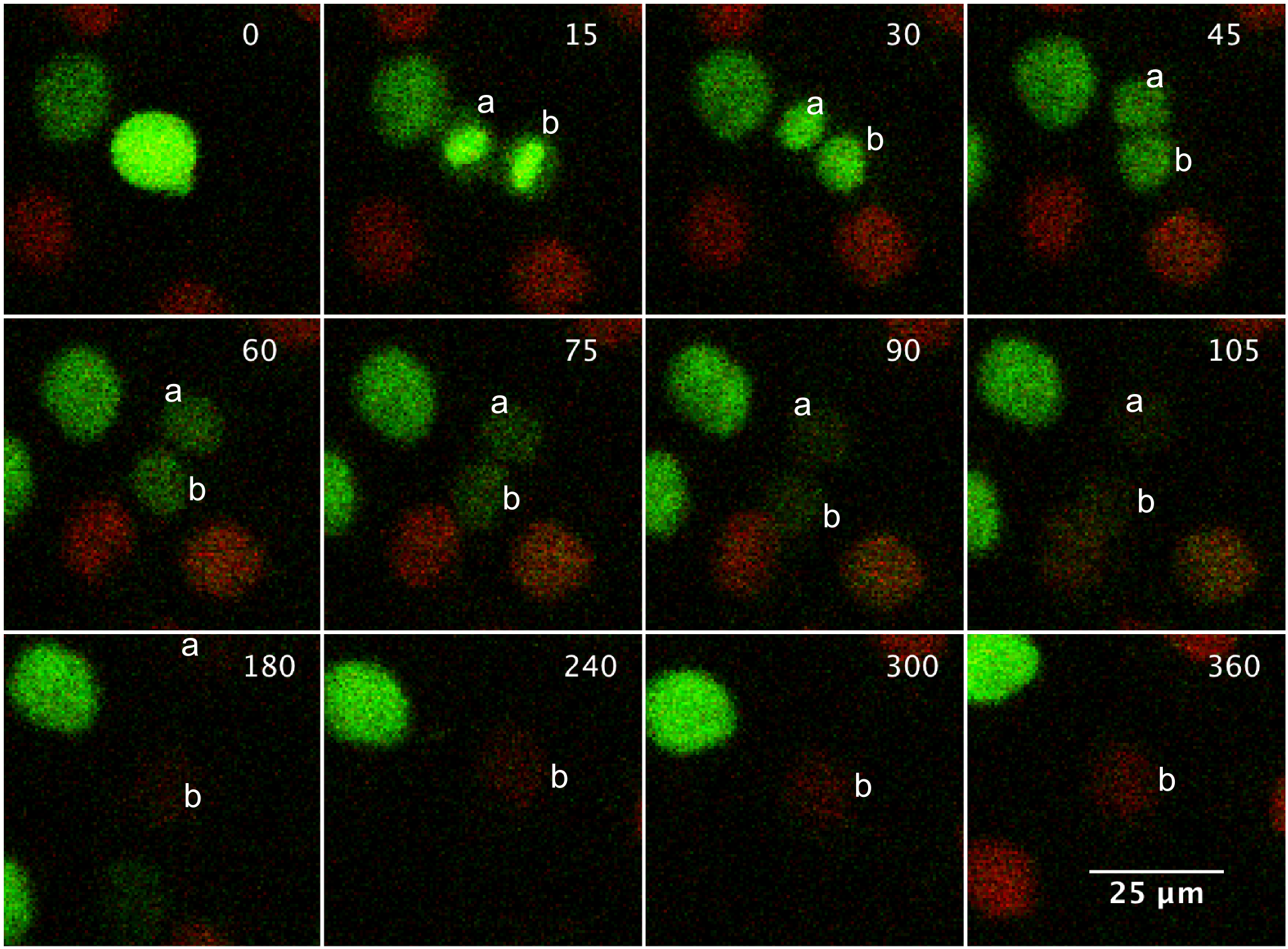

Supplement: Figure S12 — Dividing cell visualised using FUCCI markers. The green FUCCI S-G2-M marker fades after mitosis followed by a slow increase in red G1 marker. Time displayed in minutes same as Figure S11 above. (TIF) [file pone.0027886.s012.tif]

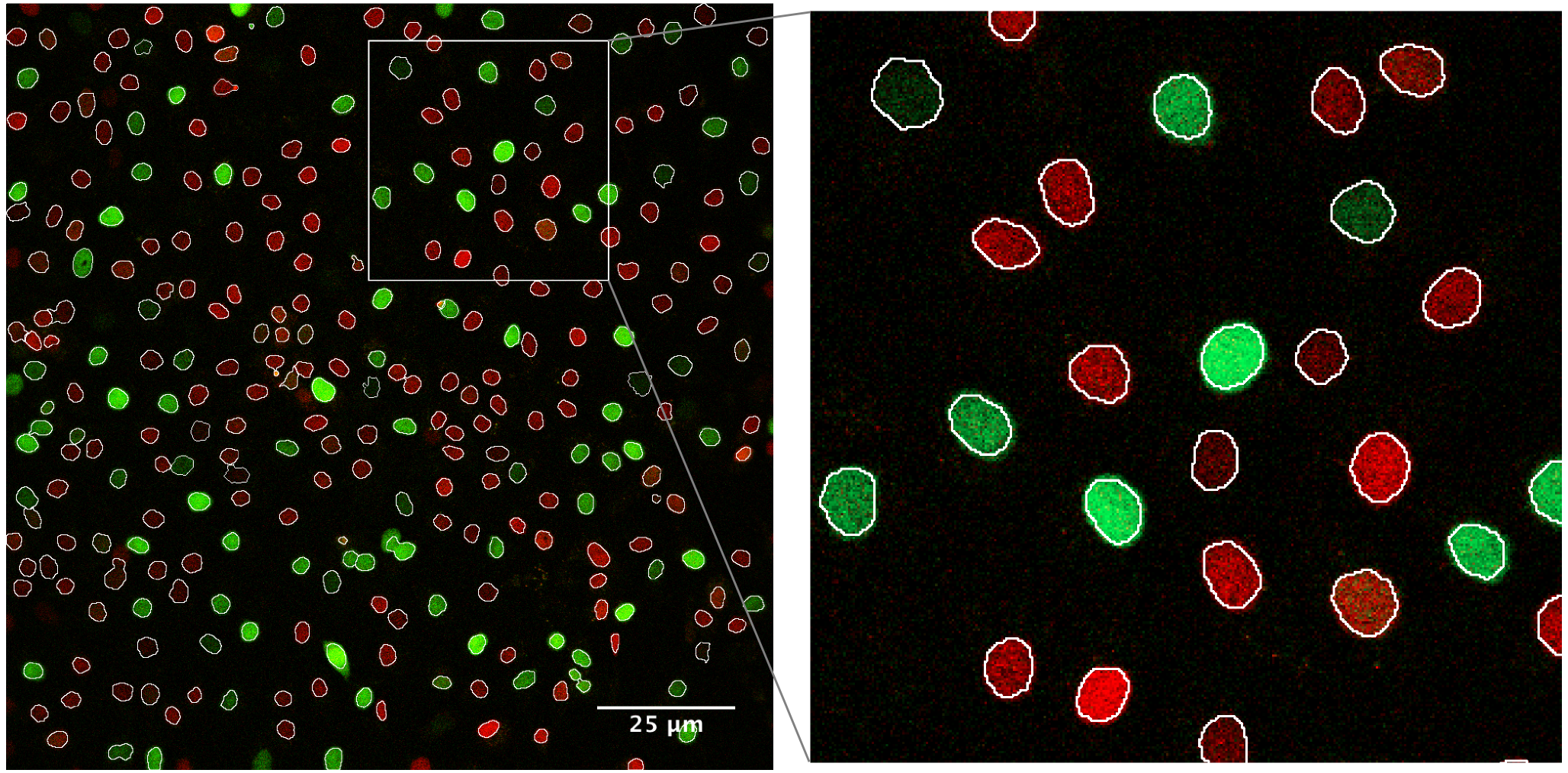

Supplement: Figure S13 — Segmentation of zebrafish PAC2 cells using the ‘Multi-Channel Segmentation’ method. (TIF) [file pone.0027886.s013.tif]
