# Supplementary material for: Extracting Fluorescent Reporter Time Courses of Cell Lineages from High-Throughput Microscopy at Low Temporal Resolution
Source: PLoS One. 2011 Dec 15;6(12):e27886. doi: 10.1371/journal.pone.0027886 (PMC3240619; doi:10.1371/journal.pone.0027886)
Supplement: Table S1 — 90–99th percentile values for change in area, frame to frame displacement during tracking, and parent-daughter distance following cell division. These values (measured in pixels) are used to select the initial threshold parameters used for tracking. (PDF) [file pone.0027886.s014.pdf]

Percentile	Change in area	Displacement	Parent-daughter distance
90	16.67	6.40	12.51
91	18.06	7.00	12.65
92	19.69	7.21	12.81
93	21.39	7.81	13.08
94	23.77	8.36	13.80
95	25.84	9.05	14.23
96	29.07	10.00	14.64
97	33.52	10.82	15.39
98	40.97	12.17	16.52
99	52.13	15.00	18.39
