# Supplementary material for: Extracting Fluorescent Reporter Time Courses of Cell Lineages from High-Throughput Microscopy at Low Temporal Resolution
Source: PLoS One. 2011 Dec 15;6(12):e27886. doi: 10.1371/journal.pone.0027886 (PMC3240619; doi:10.1371/journal.pone.0027886)
Supplement: Table S2 — Tracking precision for zebrafish PAC2 cells visualised using FUCCI markers [39]–[41]. The segmentation and tracking adjustments represent the percentage of frames which required manual intervention to preserve accurate tracking. The longest continuous sequence was observed with cell 8 at over 50 hours without corrections. Following division, daughter cells fade to close to background intensity requiring cells to be manually segmented. (PDF) [file pone.0027886.s015.pdf]

Cell ID	*Lineage Length (Frames)*	*Segmentation Adjustments*	*Tracking Adjustments*	*Longest Continuous Sequence*
1	203	18.2%	11.8%	15.8%
2	68	4.4%	5.9%	48.5%
3	408	6.6%	4.4%	20.8%
4	485	8.5%	7.2%	13.8%
5	586	9.4%	6.0%	18.6%
6	425	3.8%	2.8%	22.8%
7	758	19.4%	6.2%	11.9%
8	477	5.7%	3.4%	42.3%
9	91	0.0%	2.2%	69.2%
