# Supplementary material for: Extracting Fluorescent Reporter Time Courses of Cell Lineages from High-Throughput Microscopy at Low Temporal Resolution
Source: PLoS One. 2011 Dec 15;6(12):e27886. doi: 10.1371/journal.pone.0027886 (PMC3240619; doi:10.1371/journal.pone.0027886)
Supplement: Text S1 — Segmentation of cell nuclei. (PDF) [file pone.0027886.s016.pdf]

Segmentation of Cell Nuclei

Because of its usually confined shape, the cell nucleus is the preferred target when it comes to segmenting or distinguishing individual cells. Also, there is significant overlap of cell bodies or cytoplasm, which is less likely to occur with nuclei. Distribution of nuclear size, details on nuclear overlap (2.8%) and oversegmentation are given in Figure S1. Ideally, fusing a nuclear localization sequence to a fluorescent protein of interest will restrict its expression and therefore its analysis to the nucleus. Many segmentation methods can be fine-tuned to work at a particular cell density but often struggle to work at different densities, as commonly encountered in time-series experiments of dividing cells. In the main text we compare the performance of different standard methods, which either involve thresholding of intensities, globally or adaptively [1], or are based on structural filters (scaling index, [2]). In the Gaussian Maxima method images are convolved with a Gaussian kernel to suppress image noise or sub-nuclear features [3]. It yields nuclear nucleus positions as local maxima, which can be used as seeds to segment the whole cell nucleus by successively incorporating neighbour pixels until their intensities fall below a certain threshold, a method known as Seeded Growth [4]. Since this method sets a threshold according to the local intensity maximum, it is regionally adaptive. Where nuclei of neighbour cells tend to be merged into a single object, standard watershed algorithms often suffice to separate individual nuclei reasonably well [5].

1. Sezgin M, Sankur B (2004) Survey over image thresholding techniques and quantitative performance evaluation. Journal of Electronic Imaging 13: 146-168.
2. Jamitzky F, Stark RW, Bunk W, Thalhammer S, Rath C, et al. (2001) Scaling-index method as an image processing tool in scanning-probe microscopy. Ultramicroscopy 86: 241-246.
3. Fenistein D, Lenseigne B, Christophe T, Brodin P, Genovesio A (2008) A fast, fully automated cell segmentation algorithm for high-throughput and high-content screening. Cytometry Part A 73A: 958-964.
4. Mehnert A, Jackway P (1997) An improved seeded region growing algorithm. Pattern Recognition Letters 18: 1065-1071.
5. Roerdink JBTM, Meijster A (2000) The watershed transform: definitions, algorithms and parallelization strategies. Fundam Inf 41: 187-228.
