# Supplementary material for: Extracting Fluorescent Reporter Time Courses of Cell Lineages from High-Throughput Microscopy at Low Temporal Resolution
Source: PLoS One. 2011 Dec 15;6(12):e27886. doi: 10.1371/journal.pone.0027886 (PMC3240619; doi:10.1371/journal.pone.0027886)
Supplement: Text S2 — Description of algorithms and parameters used for segmentation. (PDF) [file pone.0027886.s017.pdf]

Description of Algorithms and Parameters used in Segmentation

Threshold Segmentation

Images were de-noised by convolving with a Gaussian kernel (σ=0.9) followed by a Rolling-ball background subtraction (r=50). For the global threshold, a different threshold value was used for each frame. A binary image was produced where pixel intensities lower than the threshold were set to zero. Finally the ImageJ watershed transform was applied to separate touching or clumped cells. Features were filtered according to size to remove any small particles or debris which may have been present.

Local threshold sementation followed the same procedure with Niblack adaptive thresholding (available with the Fiji distribution of ImageJ) applied to each frame.

Seeded Growth

The Gaussian Maxima/Seeded Growth method consists of two sections. The first identifies the centres of nuclei which are used as 'seeds' in the second part to grow the nuclei [1]. A Gaussian blur (σ=2) is applied to the image and the centres of nuclei are detected by locating local maxima using the ImageJ [2] maxima finder.

The seeded growth operation takes a threshold value and a maximum size limit for the growing cells. The threshold is multiplied by the peak intensity for each maximum and growth continues until the intensity falls below this value. The image produced is a mask which is the same size as the original image, where a pixel value of zero represents background and a non-zero number represents the cell ID.

Each cell seed is represented by a centre position and a set of vectors (angle and length) which point to the nucleus outline. When the cells grow, the points on the outline become more separated. If the distance between adjacent points exceeds a set value a new interpolated point is inserted with angle and length calculated as the arithmetic mean of the neighbouring vectors.

Multi-Channel Segmentation

The seeds are identified using the Gaussian Maxima process described above. Nucleus outlines are grown using a variant of the seeded growth with an additional 'colour' component. The colour difference between pixels is calculated as the distance between two normalized vectors consisting of the intensities of the image channels. Growth continues as before with the additional constraint of the colour difference which prevents two cells of similar greyscale intensity but different colour from growing together (See Figure S13).

Scaling Index segmentation

After background subtraction the scaling index method was run with radii r_1=3 & r_2=9 [3]. The nuclei appeared as low dimensional objects. Each frame was thresholded, followed by the Watershed transform to separate touching cells. The resulting segmentation mask was filtered according to size using the same method employed in the threshold segmentation.

CellProfiler

The images were convolved with a Gaussian kernel (σ=1 pixel) followed by background subtraction (block size=50 pixels, polynomial smoothing).

Cells were identified using the Background Adaptive threshold method. Clustered cells were split apart using the 'Shape' method which uses a distance-transform of the binary image to detect 'clumped' objects.

1. Fenistein D, Lenseigne B, Christophe T, Brodin P, Genovesio A (2008) A fast, fully automated cell segmentation algorithm for high-throughput and high-content screening. Cytometry Part A 73A: 958-964.
2. Rasband WD (1997-2010) U. S. National Institutes of Health, Bethesda, Maryland, USA. U. S. National Institutes of Health, Bethesda, Maryland.
3. Jamitzky F, Stark RW, Bunk W, Thalhammer S, Rath C, et al. (2001) Scaling-index method as an image processing tool in scanning-probe microscopy. Ultramicroscopy 86: 241-246.
