# Supplementary material for: Extracting Fluorescent Reporter Time Courses of Cell Lineages from High-Throughput Microscopy at Low Temporal Resolution
Source: PLoS One. 2011 Dec 15;6(12):e27886. doi: 10.1371/journal.pone.0027886 (PMC3240619; doi:10.1371/journal.pone.0027886)
Supplement: Text S3 — Description of LineageTracker software user interface. (PDF) [file pone.0027886.s018.pdf]

Software Minimum Requirements

- Fiji or ImageJ version 1.44o or newer
- Image5D (pre-installed with Fiji)
- Java 1.6 or newer
- 32 or 64 bit Windows, OSX or Linux.
- 2Gb RAM

Segmentation User Interface

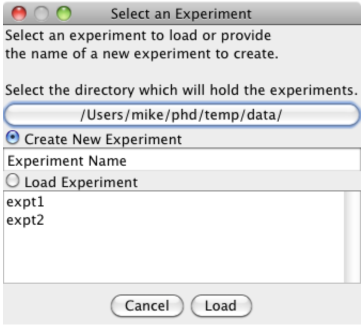

Select the folder which will hold the experiments, such as the 'data' directory below. Either select an experiment name or type in the name of a new experiment. The data is stored in the directory structure below.

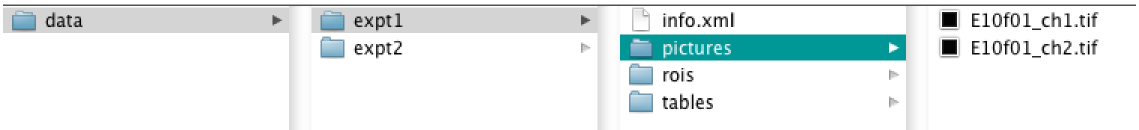

Experiment directory structure. The info.xml file describes the experiment. The 'rois' and 'tables' directories are filled when the experiment is analysed.

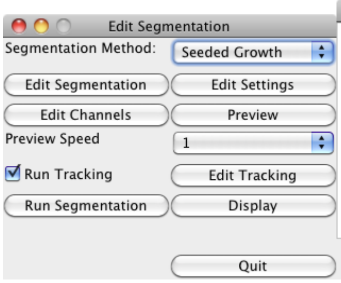

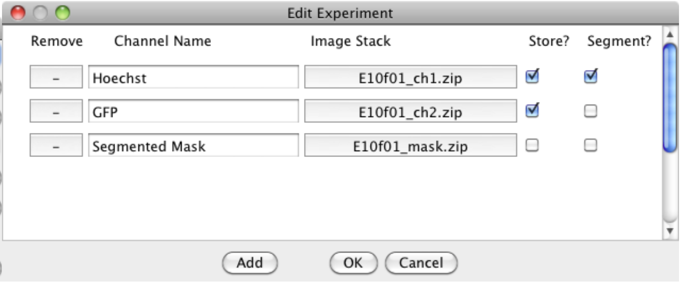

**Control**	**Function**
Edit Experiment Window:	
Add	Adds a new image channel. Images must be uncompressed TIFF stacks.
[-]	Remove the image channel
Store	Store intensity information for this channel
Segment	Use this channel in the segmentation algorithm
Edit Segmentation Window:	
Segmentation Method	Select the segmentation method.
Edit Segmentation	Edit method-specific segmentation parameters.
Edit Settings	Experiment-specific segmentation parameters including maximum & minimum object size and background subtraction.

Edit Channels	Opens the 'Edit Experiment' window.
Preview	Displays a window previewing the segmentation.
Edit Tracking	Edit the tracking parameters
Run Segmentation	Run the segmentation (and tracking if required)
Display	Call the Tracking Viewer

Tracking View User Interface

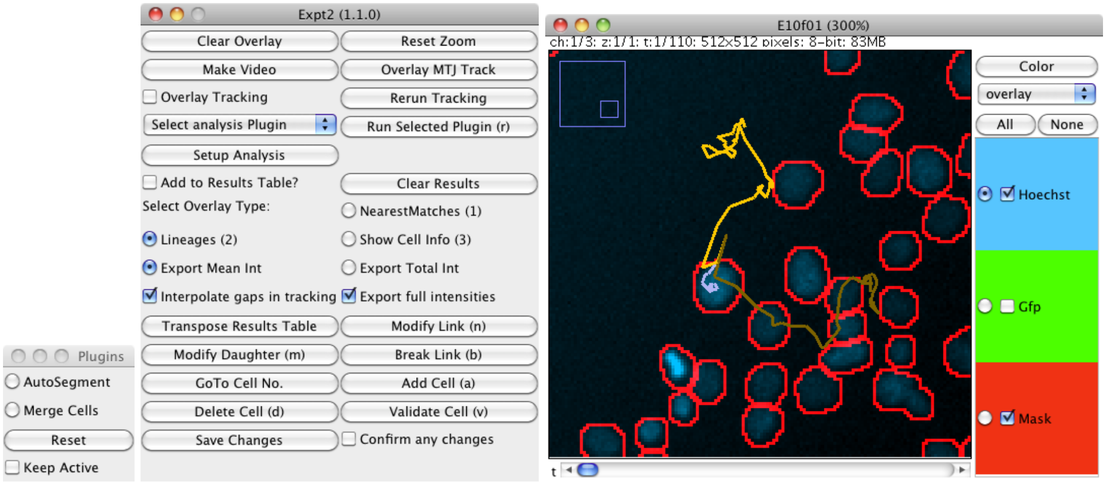

Tracking viewer running as an ImageJ plug-in, displaying the trajectory of a tracked cell. The plugin selector window allows rapid switching between data analysis or editing plugins.

Control	Function
Control Panel Window:	
Clear Overlay	Remove any overlaid highlighting from the window.
Reset Zoom	Reset to 100% magnification.
Make Video	Create a image time-series complete with highlighted cells and tracks.
Load MTJ Track	Load and overlay an MTrackJ trajectory.
Overlay Tracking	When a tracked cell is selected, draw the full trajectory from current timepoint onwards.
Rerun Tracking	Allows tracking to be run with a different method or parameters.
Select Analysis Plugin	If any additional analysis plugins have been installed, they can be selected from here. When a cell is clicked in the viewer window, the plugin will be called for that cell.
Do Analysis	Call the selected analysis plugin for all cells in the experiment.
Setup Analysis	Edit any settings (if needed) for the analysis plugin. Also used to display a brief description of the plugin.
Add to Results Table	When a cell is selected, add fluorescence intensity data to an ImageJ Results Table
Clear Results	Clear any currently open Results Tables.
Overlay Types: the next 3 buttons control the information displayed or added to a results table when a cell is highlighted in the window.	

1) Nearest Matches	Overlay the Movement Scores for trajectories leaving the highlighted cell.
2) Lineages	Display tracking, including daughter cells.
3) Cell Info	Display fluorescence information for current timepoint.
Export Total/Mean Int	Selects between exporting the integrated or mean cell intensity to the results table.
Interpolate Gaps	Interpolates the intensity if tracking skips a frame.
Export Full Intensities	ON: Exports the full lineage intensity for daughter cells<br>OFF: Only export intensities from division onwards for daughter cells.
The next three buttons modify tracking based on the previous two highlighted cells. To alter the link between two cells (in adjacent frames), click on one cell then move to the next frame and click on the second cell. Finally select one of the following buttons.	
Modify Link	Add the two selected cells as a trajectory
Modify Daughter	Add a trajectory, marking it as a cell division. If the parent cell already has a tracked 'next cell', that is marked as the other daughter cell.
Break Link	Remove the tracking between the two selected cells.
Go To CellID	Select a cell based on its ID number
Save Changes	Save any segmentation or tracking changes.
Add Cell	Expects a 'closed' ROI (such as oval or polygon). Calculates the cell features and adds a new cell.
Delete Cell	Deletes the currently selected cell.
Validate Cell	Marks the currently selected trajectory as 'validated'. The validated cells are drawn with filled-in masks for easy identification.
Confirm Changes	Prompts the user before any actions which change the cell data.
Cell Image Window	
The tick-boxes show or hide the following image channels.	
Time t	The primary fluorescence channel
Mask	Segmentation outlines
GFP	Any additional fluorescence channels.
Plugins Window	
This window lists all available 'Analysis Plugins' and allows easy selection between them. Two plugins are currently available.	
AutoSegment	After selecting this and clicking on a cell in the image window, the plugin will attempt to calculate the outline of the cell and add it to the experiment data. A dialog box appears allowing the segmentation parameters to be adjusted.
Merge Cells	Requires an ROI to be drawn in the image window. When 'Run Selected Plugin' is clicked, any cells within the ROI will be merged together.
Reset	Un-selects the current selected plugin.
Keep Active	If un-selected, the current plugin will be used for the next click in the cell image window. Selecting this will allow the plugin to be run several times on different cells.

Benchmarking Software

The segmentation benchmarking consists of an ImageJ macro which receives the segmentation mask and compares the cell positions with a manually-produced 'gold standard'. The tracking benchmark software is a plugin for the LineageTracker application which requires a manually validated cell lineage. This software will be available to download from the LineageTracker website[1].

[1] http://go.warwick.ac.uk/lineagetracker
